# Transcriptome analysis of human brain microvascular endothelial cells response to *Neisseria meningitidis* and its antigen MafA using RNA-seq

**DOI:** 10.1038/s41598-019-55409-y

**Published:** 2019-12-10

**Authors:** Evelína Káňová, Zuzana Tkáčová, Katarína Bhide, Amod Kulkarni, Irene Jiménez-Munguía, Patrícia Mertinková, Monika Drážovská, Punit Tyagi, Mangesh Bhide

**Affiliations:** 1 Laboratory of Biomedical Microbiology and Immunology, The University of Veterinary Medicine and Pharmacy, Komenskeho 73, 04001 Kosice, Slovakia; 2 Institute of Neuroimmunology of Slovak Academy of Sciences, 84510 Bratislava, Slovakia

**Keywords:** Infectious diseases, Pathogens

## Abstract

Interaction of *Neisseria meningitidis* (NM) with human brain microvascular endothelial cells (hBMECs) initiates of multiple cellular processes, which allow bacterial translocation across the blood-brain barrier (BBB). NM is equipped with several antigens, which interacts with the host cell receptors. Recently we have shown that adhesin MafA (UniProtKB-X5EG71), relatively less studied protein, is one of those surface exposed antigens that adhere to hBMECs. The present study was designed to comprehensively map the undergoing biological processes in hBMECs challenged with NM or MafA using RNA sequencing. 708 and 726 differentially expressed genes (DEGs) were identified in hBMECs exposed to NM and MafA, respectively. Gene ontology analysis of the DEGs revealed that several biological processes, which may alter the permeability of BBB, were activated. Comparative analysis of DEGs revealed that MafA, alike NM, might provoke TLR-dependent pathway and augment cytokine response. Moreover, both MafA and NM were able to induce genes involved in cell surface modifications, endocytosis, extracellular matrix remodulation and anoikis/apoptosis. In conclusion, this study for the first time describes effect of NM on the global gene expression in hBMECs using high-throughput RNA-seq. It also presents ability of MafA to induce gene expression, which might aid NM in breaching the BBB.

## Introduction

*Neisseria meningitidis* (NM, meningococcus) causes life-threatening meningitis and fatal sepsis^1,2^. Meningococcus can successfully invade the CNS by crossing the blood-brain barrier (BBB) via transcellular (transport across the cells; transcytosis) or paracellular routes (crossing through the intercellular space without disrupting the cell structure)^3–5^. The BBB is intrinsic structure, which at its luminal side is lined by the brain microvascular endothelial cells (hBMECs)^6^. hBMECs forms continuous endothelial barrier due to the presence of tight junctions localized at the apical end of inter-endothelial space and adherens junctions localized at the basolateral endothelial cell membrane, which stabilize tight junctions^7^. The meningococcal transcytosis in the hBMECs is initiated by the formation of the membrane protrusions surrounding of bacteria^8^. The actuated process of transcytosis subsequently triggers multiple signaling cascades in the host cells, mainly by activation of β2-adrenoreceptor and β-arrestin, which leads to the organization of cytoplasmic molecular complexes by recruitment of molecular linkers ezrin and moesin (also known as ERM [ezrin-radixin-moesin] proteins)^9,10^, along with accumulation of certain membrane-integral proteins such as CD44 and intracellular adhesion molecule - ICAM-1^9,11^.

Some events in the paracellular way of the transport of meningococci are also described in which recruitment of the polarity complex Par6/Par3/PKCζ to the site of meningococcal adhesion is pivotal. Under normal circumstances, polarity complex plays a crucial role in the formation of intercellular junctions of hBMECs, however under meningococcal influence recruited polarity complex causes re-routing of proteins involved in the formation of endothelial adherens and tight junctions (e.g. VE-cadherin, β-catenin, claudin-5 *etc*.). Relocalization of junctional proteins to the place of adhesion of meningococci leads to the opening of intercellular space allowing the passage of NM through the endothelial layer^12^.

Aforementioned membrane protrusions (also called as docking structures) around *Neisseria* resemble the structures formed during the transendothelial migration of leukocytes. The protrusions are rich in filamentous (F)-actin that surround transmigrating leukocytes. It was shown that assembly of F-actin, the driving force to induce protrusions, needs the activation of small GTPases, RhoG and Rac1^13^. A massive redistribution of vascular cell adhesion molecule 1 (VCAM-1) and ICAM-1 and −2, together with the recruitment of activated ERM proteins leading to the cortical actin polymerization and cytoskeletal reorganization is found in the generation of protrusions^14,15^. The function of the membrane protrusions is to provide assistance for migrating leukocytes^16^. Pathogens such as NM might mimic initial events in the leukocyte transmigration and use docking structures to resist shear stress (caused due to the blood flow) until the creation of intracellular vacuoles.

Meningococcus expresses several surface proteins on its surface that are capable of inducing the transmigration across the endothelial layer. For example type IV pili induce signaling events that initiate transcellular passage^12^, opacity-associated protein c (Opc) interacts with cytoskeletal α-actinin, which has an impact on the modulation of various signaling pathways and cytoskeletal functions enabling meningococci to translocate across endothelial layer^17^, whereas Opa of *Neisseria gonorrhoeae* binds to the epithelial CD66 receptor and mediates tight contact leading to the transepithelial traversal^18^. In addition to these three surface proteins, meningococcus expresses several adhesins such as NadA^19,20^, MafA^20,21^, MafB^22^, major outer membrane protein P.IB^23^ and lipoproteins^20^. Here, members of Maf (multiple adhesin family) are of particular interest. MafA, encoded by the *mafA* gene, was first described as a glycolipid-binding 36-kDa protein^21^. *mafA* gene is located on *maf* genomic island present only in pathogenic *Neisseria* species^22^. Two percent of the genome of pathogenic species of *Neisseria* consist of *maf* genes^22^. It is noteworthy that, MafA is one of the principal components of outer membrane vesicles (OMVs) released by several neisserial strains^24,25^. It was previously proposed that since MafA binds cellular glycolipid such as GgO_3_ and GgO_4_^21^, it could mediate attachment of *Neisseria* or OMVs to eukaryotic cells via an as-yet-unknown receptor^26^. The binding ability of MafA to the hBMECs was confirmed recently by us with ELISA and immunocytochemistry^20^.

High-throughput RNA sequencing (RNA-seq) technology is being extensively used to analyze the transcriptomes with extreme accuracy. In contrast to the microarray, RNA-seq provides higher sensitivity and can measure gene transcripts in a greater dynamic range. Hitherto, RNA-seq has been used to reveal host responses against various infections^27–33^. As the RNA-seq is not limited to the number of probes spotted on a chip, like in hybridization techniques, a global picture of gene expression can be obtained. Understanding the complete map of gene changes underlying the initial stages of pathogen translocation across the endothelial lining of BBB could help to develop potential therapeutic and prevention strategies against rapidly progressing infectious such as NM.

This work was aimed at elucidating complete picture of the signaling events triggered by pathogenic NM in hBMECs and compare those events with gene expression evoked by MafA using RNA-seq technology.

## Results

### RNA-seq on hBMECs exposed to NM and MafA

RNA-seq was employed to understand the molecular events occurring in hBMECs during the NM invasion. Further, it was examined whether neisserial ligand - MafA produces cell response in hBMECs that could aid in the process of neuroinvasion. Steps in the production of recombinant MafA are presented in supplementary information Fig. S1 online. RNA isolated from hBMECs culture either infected with NM or exposed to recombinant MafA was assessed for its quality and the results are presented in supplementary information Fig. S2 online. Nine cDNA libraries were prepared from three biological replicates of hBMECs incubated with infectious NM (NM1 to NM3), recombinant MafA (MafA1 to MafA3) and hBMECs without any exposure (NC1 to NC3). All libraries had optimal fragment size between 150–300 nt (Supplementary information Fig. S3 online). Sequencing yielded 12 million raw reads per sample in case of hBMECs exposed to NM, while in case of hBMECs induced with MafA average reads were 9 million (Supplementary information Table S1 online). In total, 11,398 genes were mapped for each sample (Supplementary dataset S1 online). Genes with a minimum average logCPM (count per million) of 3 were considered to be differentially expressed and were included in the differential expression analysis. Genes with logFC (fold change) ranging beyond ± 1.2 were included in the final list of DEGs (Supplementary information Fig. S4 online). The *p*-value for shortlisted DEGs was checked and any gene with p > 0.01 was removed. Raw RNA-seq data and processed data showing DEGs can be downloaded from EBI Arrayexpress repository (https://www.ebi.ac.uk/arrayexpress/) deposited under assesion number E-MTAB-8008.

### Differentially expressed genes and validation

**Figure 1:**
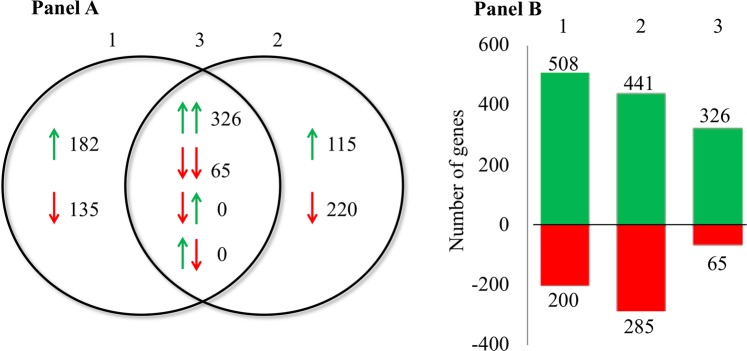
Graphic presentation of the differentially expressed genes (DEGs). (Panel A) Venn diagram presenting number of DEGs in hBMECs incubated with *Neisseria meningitidis* (1), MafA (2) and common in both treatments (3). Green arrows – upregulated DEGs. Red arrows – downregulated DEGs. (Panel B) Bar graph showing up (green) and downregulated (red) DEGs in each treatment. DEGs in hBMECs incubated with *N. meningitidis* (1) or MafA (2). 3 – common genes present in both treatments.

In total 708 genes were differentially expressed in hBMECs undergoing NM infection, whereas 726 genes were found to be differentially expressed in MafA exposed cells (Supplementary dataset S2 online). Among the 708 DEGs, 508 genes were upregulated and 200 genes were downregulated. On the other hand, from 726 DEGs, 441 genes were upregulated and 285 genes were downregulated. When searched for consensus entries, 391 genes were observed to be common in both treatments (NM vs. MafA − 326 upregulated and 65 downregulated common genes) (Fig. 1). To test the specificity of cell signalling cased due to MafA, non-related ligand of *Streptococcus pneumoniae* was used as a control to induce hBMEC keeping same conditions (e.g. 6 hrs induction time, 1 nMol protein concentration/treatment). Non-related protein evoked only 113 DEGs compared to 726 DEGs in case of MafA (Supplementary dataset S2 online). Only 87 DEGs were common between two treatments, in which all common DEGs were upregulated (Supplementary dataset S2 online and Supplementary information Fig. S5 online). One DEG (GALNT15) was downregulated in the case of MafA (logFC −1.74), while its expression was increased (logFC 1.42) when cells were induced with the non-related ligand. It is important to underline here that 639 DEGs were evoked only in case of MafA, which indicate that the signalling events occurred in the hBMECs might be specific and not because of non-specific induction of the cells, which could be observed due to the use of exogeneous recombinant protein.

**Figure 2:**
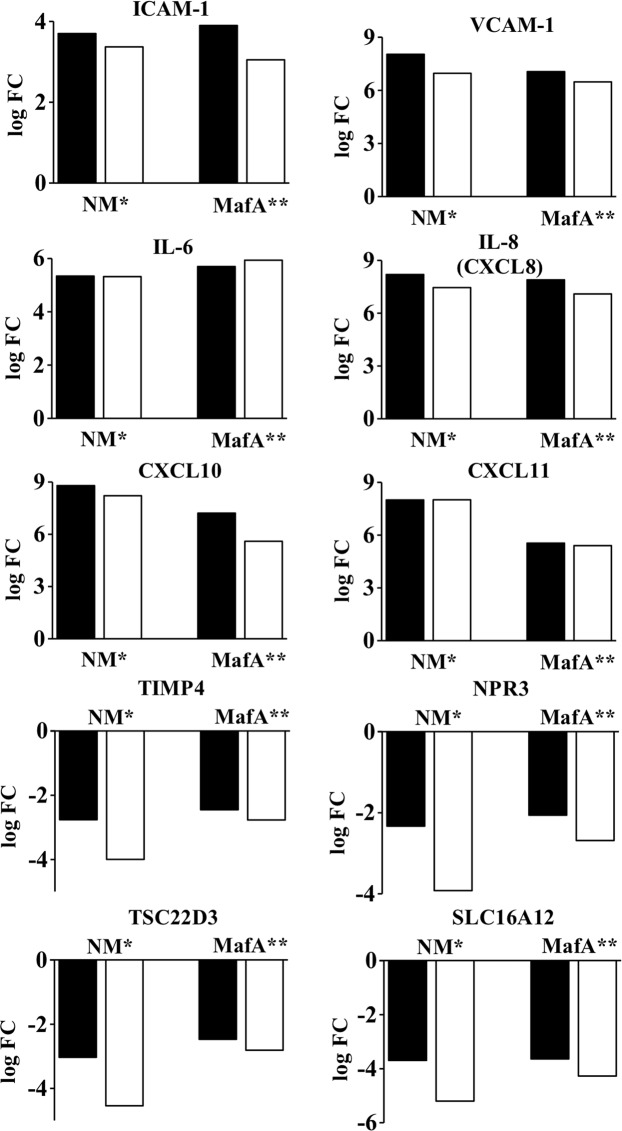
Validation of DEGs with qRT-PCR. hBMECs induced by *Neisseria* (NM) or MafA (MafA). Black bars - logFC values from RNA-seq, white bars - logFC calculated from qRT-PCR. *Pearson correlation coefficient (PCC) r = 0.9966; P-value < 0.01; ** PCC r = 0.9959, P-value < 0.01. Please note that, standard deviation (SD) is not shown here as the expression level (log² of ΔΔCT) of DEGs in qRT-PCR was calculated based on average CT values of triplicates (the SD of CT were ranged between 0.005 to 0.28).

**Figure 3:**
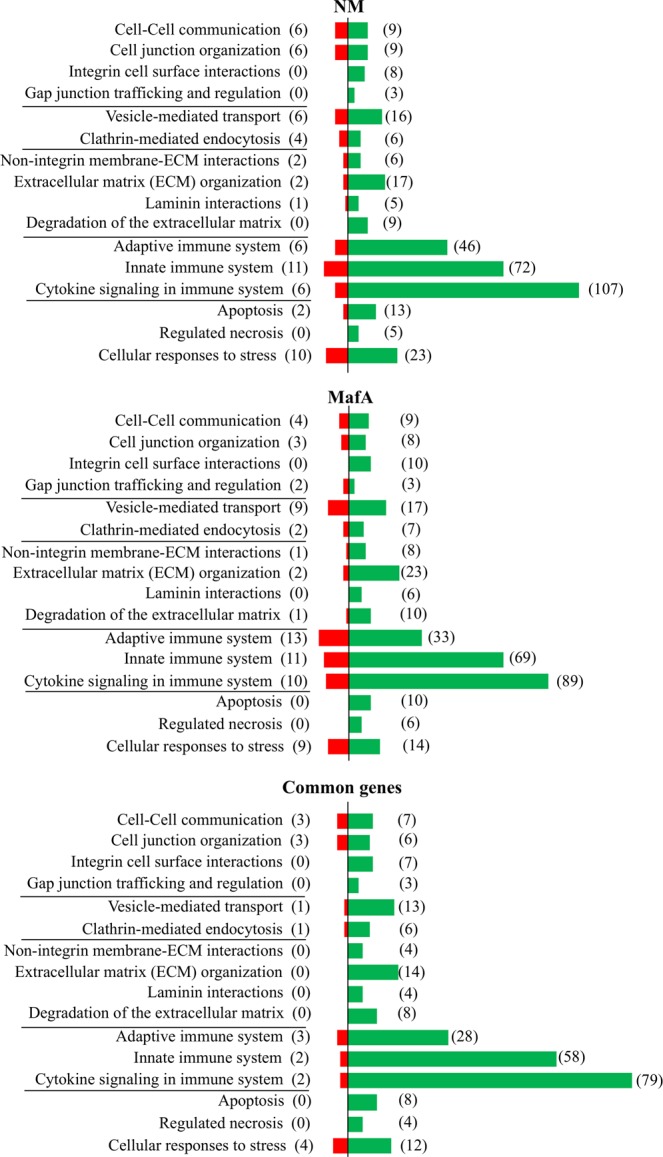
Segregation of the DEGs according to the GO biological processes. DEGs from hBMECs incubated with *N. meningitidis* (NM) or protein MafA (MafA). Common genes are the DEGs present in both treatments. Reactome server was used to group the DEGs according to GO biological processes. Only the GO biological processes associated with translocation of pathogen across BBB are presented here. Green bars – upregulated DEGs. Red bars – downregulated DEGs. Number of DEGs is displayed in parenthesis.

**Figure 4:**
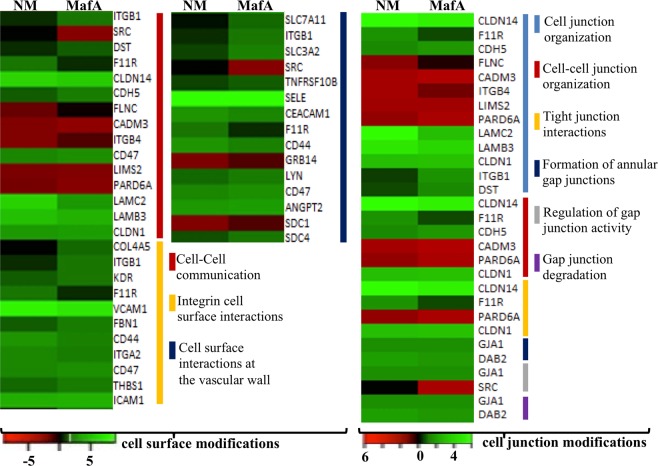
Comparative analysis of GO biological processes activated by NM and MafA in hBMECs. Heat maps showing top biological processes related to the cell surface modifications and cell junction modifications are presented. Green shaded genes - upregulated, red shaded genes - downregulated. Shading intensity indicates the degree that each gene was upregulated or downregulated. Black color indicates no significant change in the expression. Range of the fold change (logFC values) is presented in the scale.

**Figure 5:**
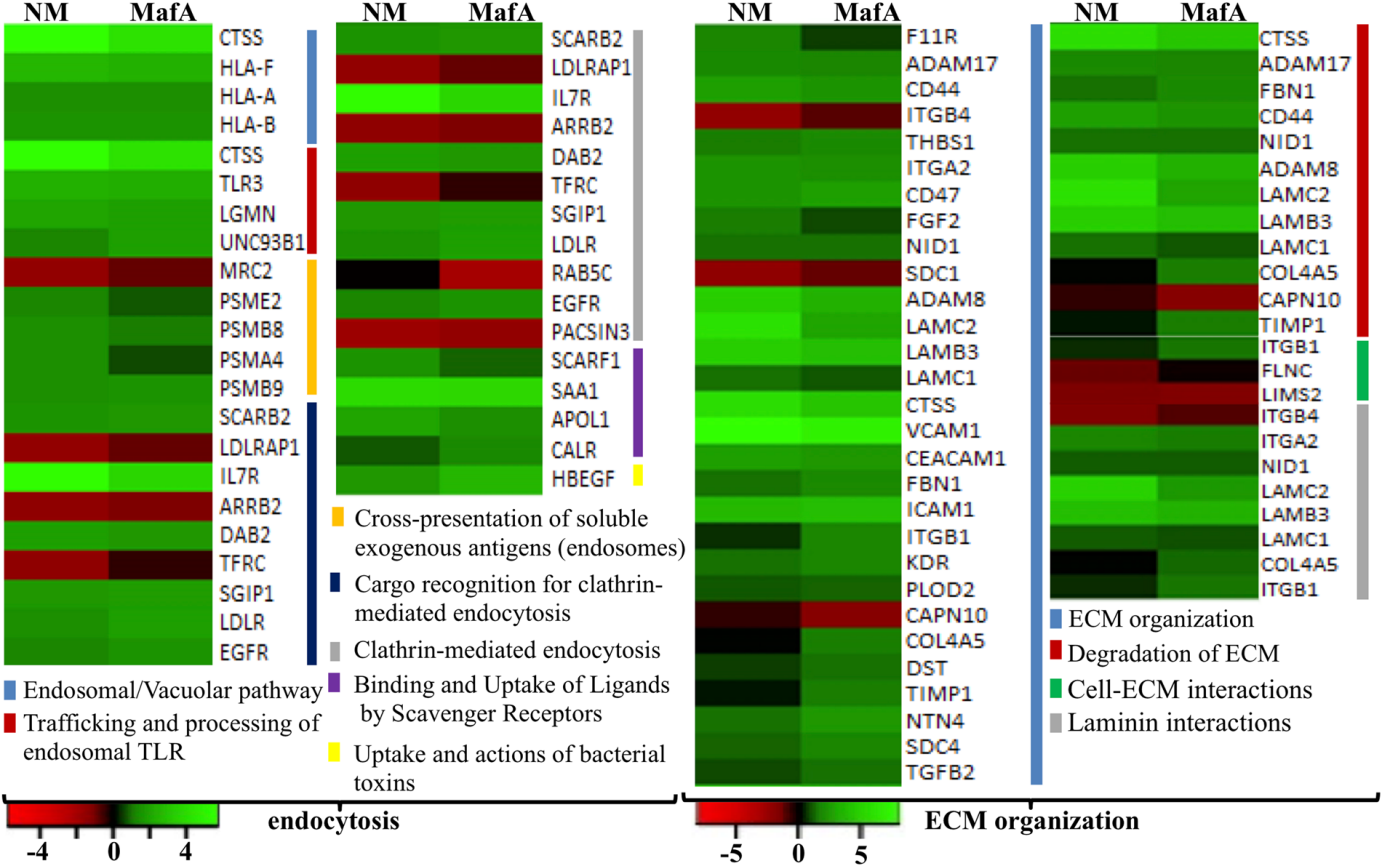
GO biological processes activated by NM and MafA in hBMECs. Heat maps showing top biological processes related to the endocytosis and ECM (extracellular matrix) organization are presented. Green shaded genes - upregulated, red shaded genes - downregulated. Shading intensity indicates the degree that each gene was upregulated or downregulated. Black color indicates no significant change in the expression. Range of the fold change (logFC values) is presented in the scale.

**Figure 6:**
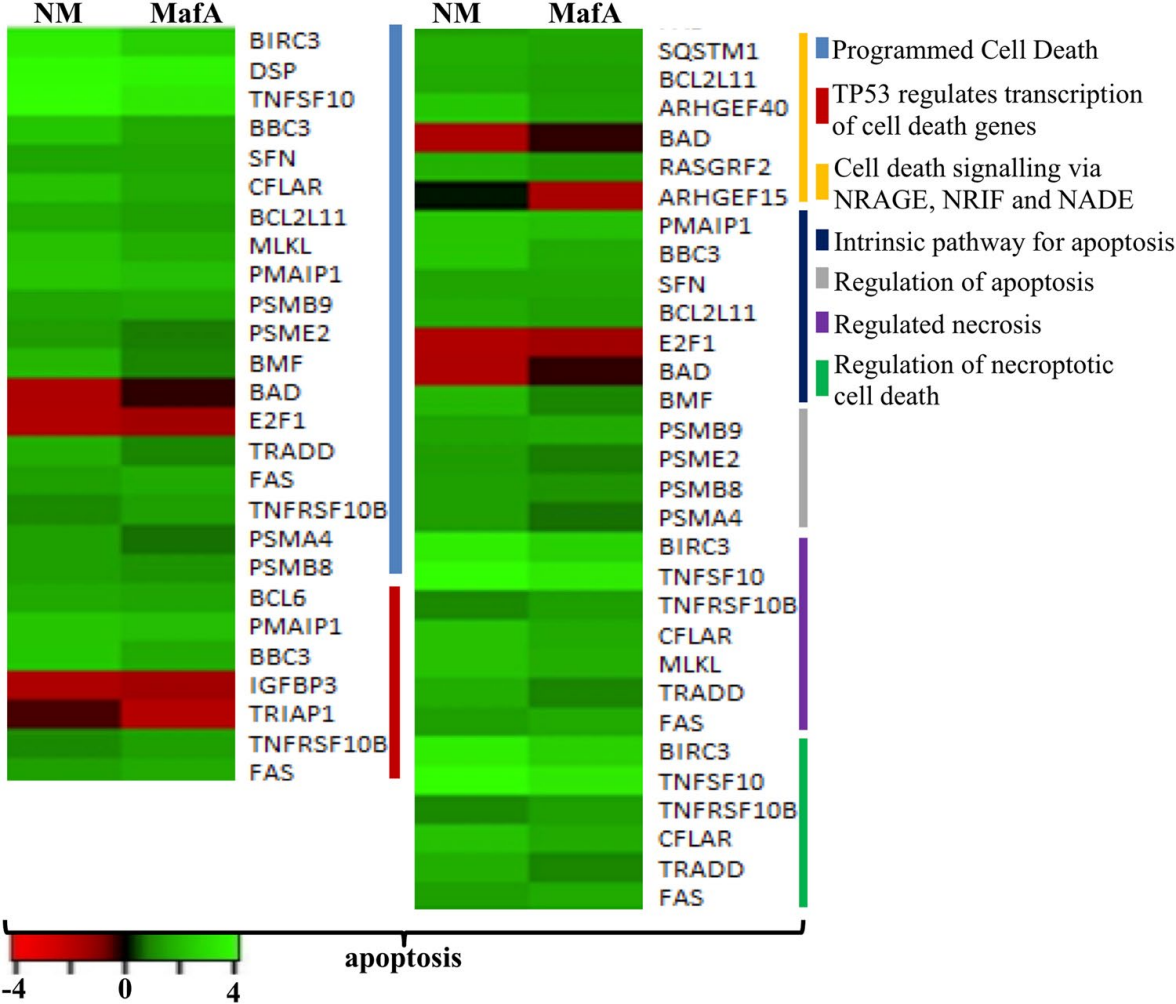
Comparison of the DEGs associated with apoptosis activated by NM and MafA in hBMECs. Heat maps showing top biological processes in apoptosis. Green shaded genes - upregulated, red shaded genes - downregulated. Shading intensity indicates the degree that each gene was upregulated or downregulated. Black color indicates no significant change in the expression. Range of the fold change (logFC values) is presented in the scale.

**Figure 7:**
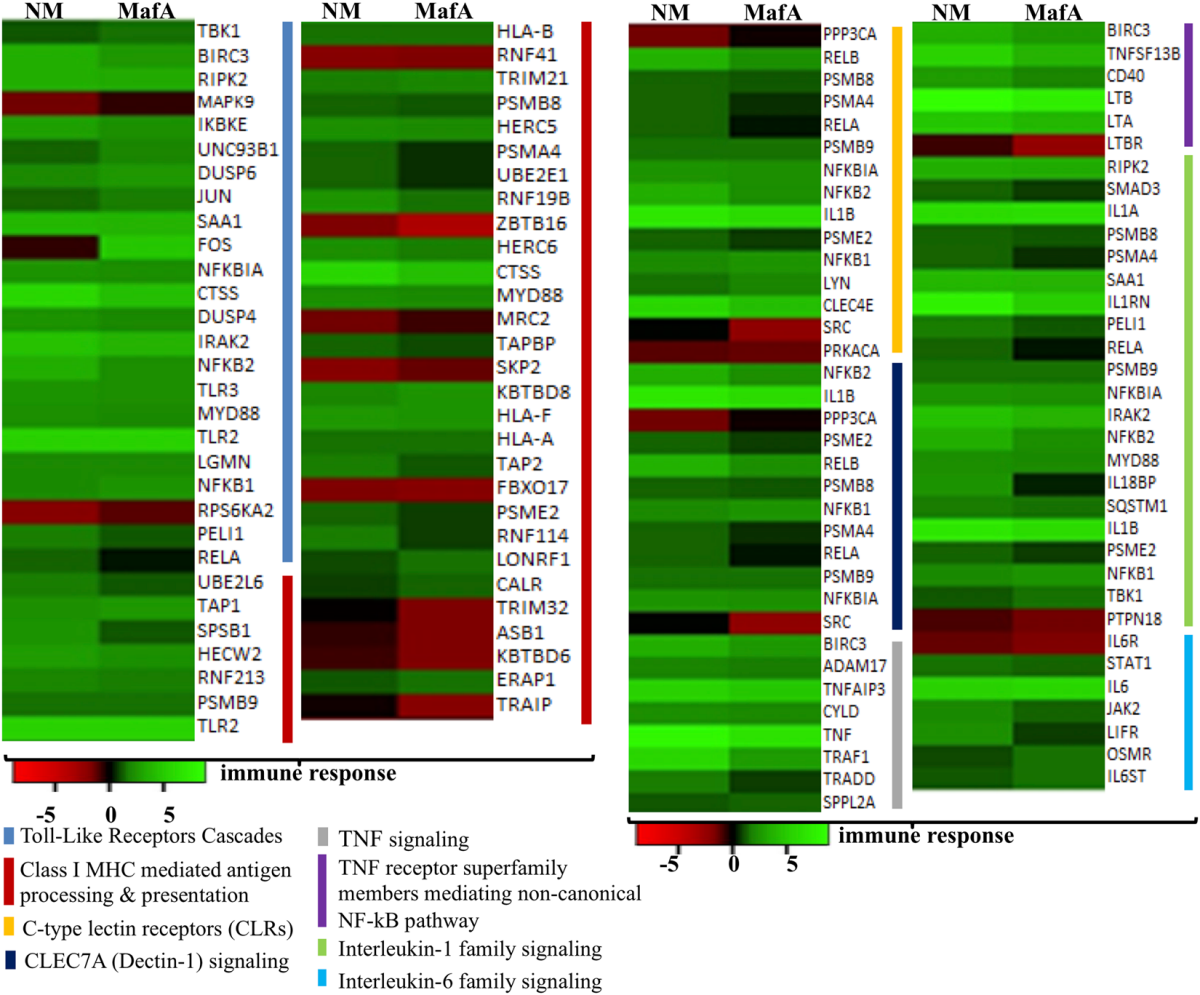
The differential expressed genes related to immune response in hBMECs activated by NM and MafA. Heat maps showing top biological processes related to the immune response. Green shaded genes - upregulated, red shaded genes - downregulated. Shading intensity indicates the degree that each gene was upregulated or downregulated. Black color indicates no significant change in the expression. Range of the fold change (logFC values) is presented in the scale.

To validate the results obtained from RNA-seq, differential expression of 10 representative genes was analyzed with qRT-PCR. Results obtained from both techniques were consistent (Fig. 2) when calculated the Pearson correlation coefficient PCC (r = 0.996 for hBMECs exposed to NM and r = 0.995 for hBMECs exposed to MafA; p < 0.01). Following the validation of the results, DEGs were segregated according to the GO biological processes using peer-reviewed server - Reactome (https://reactome.org/)^34^ (Fig. 3 and Supplementary dataset S3 online). Later, we focused on the GO biological processes which may play a role in the neisserial translocation across the BBB (e.g. cell surface modification, cell junction modification, endocytosis, extracellular matrix organization etc.; Figs. 4–7). In the following sections, these biological processes are described in details.

### Expression of DEGs involved in cell surface modifications

Multiple DEGs were enriched in three GO biological process related to cell surface modification *viz*. “cell-cell communication (15 DEGs)”, “integrin cell surface interactions (11 DEGs)” and “cell surface interactions at the vascular wall (15 DEGs)”. Among these DEGs, E-selectin (*SELE*) was substantially induced in hBMECs incubated with both NM and MafA (NM logFC 8.89, MafA logFC 8.74) (Fig. 4, Supplementary dataset S2 online). Other adhesins like CEACAM, ICAM-1, VCAM-1, CD44 and CD47 were also upregulated in both cases, whereas junctional adhesion molecule 1 (F11R or JAM-1) was evoked only in case of NM (Fig. 4). Relevance of junctional proteins lies in their ability to relocate underneath neisserial attachment and to form membrane protrusion surrounding the bacterial colony. This leads to the weakening of the intercellular junctions facilitating translocation of NM via paracellular route. ICAM-1, VCAM-1 and CD44 are known to bind ERM proteins, which help in the polymerization of cortical actin and cytoskeletal reorganization. DEGs related to the cell junction modification were grouped into 6 GO biological processes (Fig. 4). Genes related to the formation of tight junction were sorted into 3 GO biological processes namely “tight junction interaction”, “cell-cell junction organization” and “cell junction organization”. CLDN1, CLDN14 and F11R are among important candidates involved in tight junction formation. Except F11R, all genes mentioned above were upregulated in hBMECs incubated with NM or MafA. Major gene involved in the formation of adherence junction, CDH5 (VE-cadherin, NM logFC 1.35, MafA logFC 1.79) was put into the GO biological process “cell-cell junction organization”. Whereas, the CADM3 belonging to the nectin family, essential for adherens junction formation and maintenance, was significantly downregulated (NM logFC −2.05, MafA logFC −2.51). In this study we also observed upregulation of candidates (GJA1 and DAB2) involve in the maintenance of gap junction (Fig. 4). Pathogens can use the gap junction channels to spread the intracellular or toxic signals to the neighbouring cells.

### DEGs involved in endocytosis

Bacteria exploit endocytosis to cross the BBB via transcellular way of pathogen transport. DEGs were grouped into seven GO biological processes - endocytosis (Fig. 5). In this GO term, it is noteworthy to observe almost similar gene expression pattern in hBMECs induced by NM and MafA. Of note, SCARB2 encoding lysosome membrane protein 2 and SGIP1 (having role in clathrin–mediated endocytosis) were upregulated. Similarly, Interleukin-7 receptor (IL7R), Serum Amyloid A1 (SAA1) and Cathepsin S (CTSS) were highly upregulated genes in this GO biological process. On the other hand, PACSIN3 (important in regulation of internalization of plasma membrane proteins), TRFC and LDLRAP1 (involved in receptor-mediated endocytosis), ARRB2 (arrestin beta 2), and MRC2 (Mannose Receptor C Type 2, mediating internalization) were downregulated in both cases. RAB5C (contributing in the process of docking and/or fusion of vesicles) was downregulated only in the cells induced with MafA (logFC −2.03).

### DEGs involved in extracellular matrix organization (ECM)

The adherence (anchoring) of the BMECs to the ECM provides integrity to the endothelial barrier. When the anchored cells are detached from the ECM substratum (a phenomenon called anoikis), integrity of BBB is compromised facilitating the entry of pathogen across BBB. The matrix metalloproteinases (MMPs) are known to be the likely effector candidates. NM might exploit anoikis as one of its translocation mechanisms. Members of the ADAM family, ADAM8 and ADAM17 were upregulated in challenged BMECs (ADAM8, NM logFC 4.72, MafA logFC 3.28; ADAM17, NM logFC 1.9, MafA logFC 1.6). ADAMs are characterized by a disintegrin and metalloprotease domains that confer adhesive properties and proteolytic activities, respectively, playing a role in the remodeling of ECM. In addition to ADAMs, two proteases namely CTSS and CAPN10 were observed in GO biological processes “ECM organization” and “degradation of ECM” (Fig. 5). CAPN10 was downregulated in the cells challenged with MafA (logFC −1.67), whereas the expression was unchanged in NM infected endothelial cells. Although the genes involved in the degradation of ECM were upregulated, simultaneous elicitation of the genes encoding structural components of basal membrane as NID1, LAMC1, LAMB3, LAMC2 (Fig. 5) was observed. COL4A5 encoding collagen type IV was induced only in hBMECs incubated with MafA. Upregulation was also found in case of the genes involved in the angiogenesis (THBS1, FGF2, NTN4), acting as cytoskeletal linkers (SDC4 and DST) and conferring the stability of collagen (PLOD2) (Fig. 5). Albeit several important upregulated genes supporting the ECM repair (mentioned above), genes encoding syndecan 1 that mediates cell binding (SDC1, NM logFC −1.96, MafA logFC –1.17) and LIM zinc finger domain 2, a member of a small family of focal adhesion proteins necessary for cell-matrix interactions (LIMS2, NM logFC −1.94, MafA logFC −2.02), were down regulated in both NM and MafA challenged cells. Another important candidate playing role in reorganizing the actin cytoskeleton, FLNC (filamin C), was down regulated only in NM challenged cells (Fig. 5).

### DEGs involved in the cell death - apoptosis

Numerous genes in hBMECs induced by NM or MafA were categorized in apoptosis signaling pathway. Majority of the genes were upregulated in the GO biological process “programmed cell death”. Several key proapoptotic genes like TNFSF10, BBC3, BCL2L11, PMAIP1, BMF, TRADD, FAS and TNFRSF10B were evoked in both cases. TRIAP1, a proapoptotic gene was downregulated in endothelial cells treated with MafA only (MafA logFC −1.80). On the other hand, BAD gene was downregulated only when cells were challenged with NM (NM logFC −1.57).

In case of tumor suppressor TP53 (P53) associated proapoptotic genes (GO biological process “TP53 regulates transcription of cell death genes”), PMAIP1, BBC3, TNFRSF10B and FAS were upregulated in case of both NM or MafA treated hBMECs. However, IGFBP3 was downregulated in both cases and TRIAP1 was downregulated only in MafA treated endothelial cells. The majority of apoptotic genes that are transcriptional targets of TP53 promote apoptosis. It was also important to look into the genes associated with induction of intrinsic pathway for apoptosis as this pathway is stimulated by the external stimuli including bacteria or toxins. Five of the seven genes associated with intrinsic apoptosis (GO biological process “intrinsic pathway for apoptosis”) were upregulated, E2F1 gene was downregulated in both cases, while BAD gene was downregulated only in the cells treated with NM (Fig. 6).

In spite of the upregulation of several proapoptotic genes in hBMECs, inhibitor of apoptosis BIRC3 was significantly evoked (NM logFC 3.48, MafA logFC 2.50).

### DEGs related to immune response

In total 83 DEGs related to innate immunity were evoked in hBMECs induced with NM, whereas in MafA treated cells there were 80 DEGs. The set of DEGs were also grouped to the adaptive immune system (NM: 52 genes, MafA: 46 genes) and cytokine signaling in immune system (NM: 113 genes, MafA: 99 genes) (Fig. 3 and Supplementary dataset S3 online).

Meningococci employ several strategies to evade host immune system, at the same time, host cells develop an immune response to block the infection. In general, the host defense includes Toll-like receptor dependent recognition of the pathogen associated molecules, cytokine release, MHC mediated antigen presentation, etc. Although hBMECs are not immune cells, the TLR dependent induction of the NF-κB, TNF signaling and MHC class I are important to maintain endothelial layer integrity. These three GO biological processes were observed in the hBMECs induced with NM or MafA (Fig. 7). Several genes involved in “Toll-like receptor cascades” (NM: 20 DEGs, MafA: 19 DEGs) were evoked. In particular, TLR2 was found significantly upregulated (NM logFC 5.33, MafA logFC 5.35). In case of MHC I related DEGs, gene encoding cathepsin S (CTSS, NM logFC 5.73, MafA logFC 4.28) was the most upregulated candidate. 36 DEGs in case of NM and 32 DEGs in MafA treatment were evoked related to “MHC class I mediated antigen processing and presentation”.

Meningococci and its toxins are potent inductors of the inflammatory response. Genes related to “TNF signaling” (Fig. 7) were upregulated in hBMECs challenged with NM and MafA. TNF, in particular, was significantly evoked (NM logFC 8.16, MafA logFC 6.63). Exaggerated inflammatory response may cause injury to endothelial cells leading to vascular leakage, leukocyte activation and leukocyte adhesion (mediated through E-selectin, ICAM-1, VCAM-1). Interleukin 1(IL-1), which dramatically increases leukocyte adhesiveness was upregulated (IL1B; NM logFC 6.91, MafA logFC 6.10). Likewise, members of “IL-1 signaling family”, except PTPN18 gene, were upregulated in NM challenged cells. In case of MafA induced cells, expression of the SMAD3, PSMA4, RELA, IL18BP and PSME2 was not evoked, whereas, PTPN18 was downregulated (logFC −1.40) (Fig. 7). Small set of the DEGs were also assigned to GO biological processes like “C-type lectin receptors (CLRs)” (NM: 14 DEGs, MafA: 11 DEGs), “CLEC7A (Dectin-1) signaling” (NM: 12 DEGs, MafA: 8 DEGs) and “interleukin-6 family signaling” (NM: 6 DEGs, MafA: 6 DEGs) (Fig. 7).

### Differentially expressed non coding RNA transcripts

Bacterial infections not only trigger expression the protein coding genes, but also evoke several non coding RNA transcripts. Although specific functions less proved, it has been realized that the non coding RNA transcripts regulate the expression of protein coding genes that are essential in the pathogenesis. In the present study, several non-coding RNA transcripts were evoked in hBMECs exposed to both NM and MafA (Supplementary dataset S4 online). Four percent of the all DEGs identified in NM induced hBMECs were non coding, while in case of MafA they were six percent. They were mainly antisense RNA (NM 12 DEGs, MafA 12 DEGs), long intergenic non-coding RNA (lincRNA, NM 12 DEGs, MafA 13 DEGs), miscellaneous RNA (MafA 3 DEG), mitochondrial RNA (only in MafA 6 DEGs), processed/unprocessed pseudogenes (NM 3 DEG, MafA 8 DEG) and Cajal body specific RNA (only in MafA 1 DEGs). It is noteworthy that 25 out of 29 non coding RNAs in NM exposed hBMECs were upregulated. On the contrary, total number of non coding RNAs in MafA exposed hBMECs were 49 and more than 50% (29) were downregulated.

Interestingly, exposure to MafA caused significant downregulation of mitochondrial tRNA (mt-tRNA) like mitochondrially encoded tRNA tyrosine (logFC –4.11), mitochondrially encoded tRNA proline (logFC –3.05), mitochondrially encoded tRNA leucine 1 (logFC –2.57) and mitochondrially encoded tRNA phenylalanine (logFC –2.05), whereas the mitochondrial rRNAs were upregulated (mt-rRNA: MT-RNR1 logFC 2.34 and MT-RNR2 logFC 2.36). It is noteworthy that, 33 non protein coding RNA transcripts observed in the present study (both in case of NM and MafA) are novel (Supplementary dataset S4 online).

### Deletion of mafA gene of NM altered gene expression in hBMECs in contrast to wild type NM (NM wt)

To confirm that the gene expression in hBMECs described above is caused by MafA, and not merely due to the cell response to exogeneous recombinant protein, *mafA* deletion mutation (NM Δ *mafA*) was constructed and hBMECs were challenged to assess gene expression.

To knock-out the *mafA*, a cassette for homologous recombination was constructed with overlap extension PCR (OE-PCR). The proper fusion of the cassette (5′-363 bp upstream of *mafA - bla* gene encoding beta lactamase − 318 bp downstream to *mafA* gene- 3′) was confirmed by gel electrophoresis and sequencing of amplicons (Supplementary information Fig. S6 online, Panel A and B). NM was transformed with the cassette and transformants were selected in the presence of carbenicillin. Replacement of *mafA* by *bla* in NM Δ *mafA* was confirmed with PCR (Supplementary information Fig. S6 online, Panel C).

**Figure 8:**
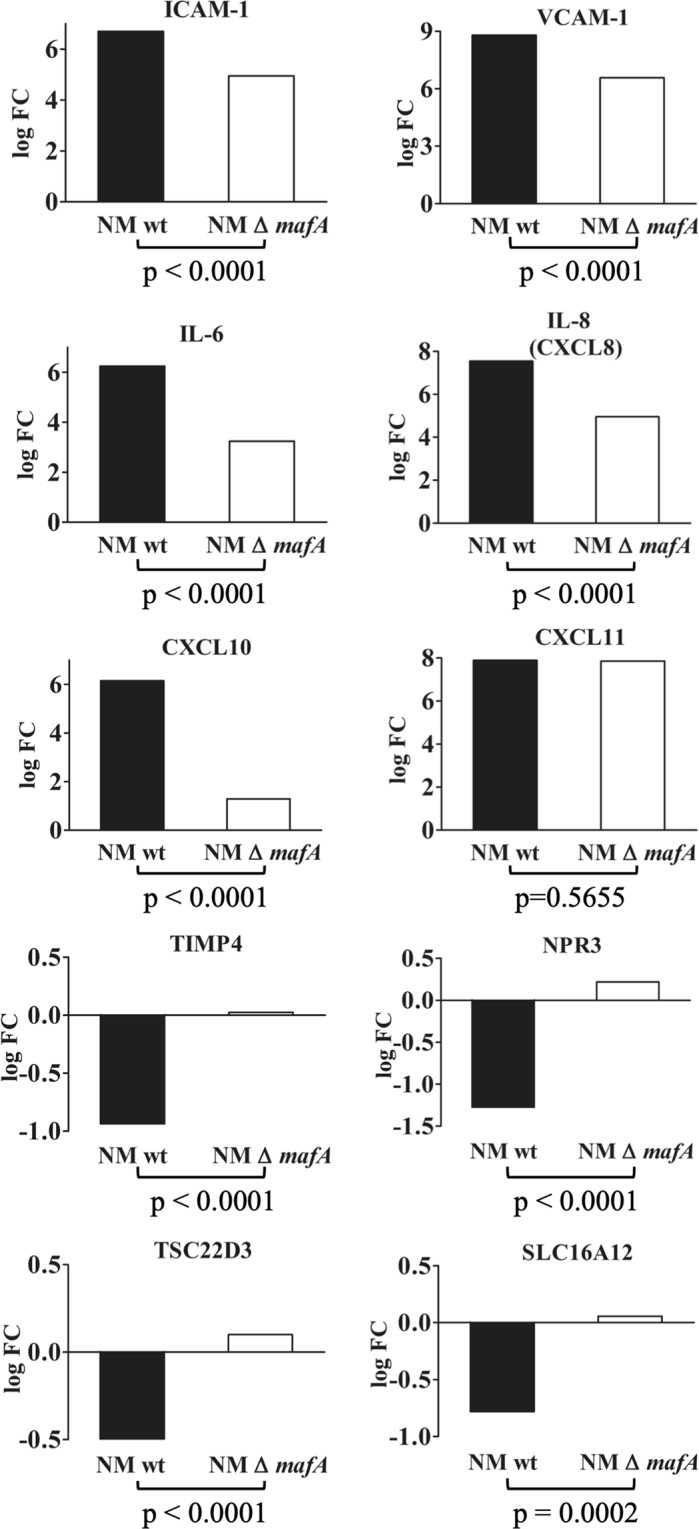
Comparison of expression of selected genes in hBMECs induced by *N. meningitidis* wild type (NM wt; black bars) and *N. meningitidis* Δ *mafA* (NM Δ *mafA*; white bars) using qRT-PCR. Statistically significant difference (p < 0.05, two-tailed p value) was calculated by unpaired *t* test. Sta*t*istics was performed with on-line statistics tool of GraphPad https://www.graphpad.com/quickcalcs/ttest1.

Among 10 representative genes analyzed, expression of ICAM-1, VCAM-1, IL-6, IL-8 and CXCL10 in induced hBMECs was significantly decreased (p < 0.0001, unpaired two-tailed *t* test, Fig. 8) in NM Δ *mafA* compared to NM wt, however, expression of CXCL11 was at the same level in case of NM Δ *mafA* and NM wt (p = 0.5655). Downregulation of TIMP4, NPR3, TSC22D3 and SLC16A12 observed in hBMECs induced with NM wt was abolished in case of NM Δ *mafA* (Fig. 8). These results indicate that MafA plays an important role in inducing the signalling events in hBMECs. It is noteworthy that, deletion of *mafA* did not abolish the differential expression of the genes (mainly upregulated), however significant reduction in the expression was observed. This might be because of other adhesins simultaneiously present on neisserial surface (like type IV pilin, Opa or Opc).

## Discussion

*N. meningitidis* evokes plethora of signaling events in the endothelial cells leading to the activation of the biological processes like formation of cortical plaques, remodulation of the cell cytoskeleton, internalization of the pathogen, overwhelming immune (inflammatory) response, dislodgement of the tight junctions, detachment of the endothelial cells (anoikis) and apoptosis^10,12,35–38^. Several surface proteins - type IV pili, Opa, Opc and MspA are shown to interact with host receptors^39,40^ and induce cell response^41,42^. However, till to date, scanty reports are available on the members of Maf family (MafA, MafB, etc.). Earlier studies on MafA like adhesin protein (gangliotetraosylceramide binding adhesin) have described this candidate as a surface adhesin that binds to the glycolipid receptors^21^, and have highlighted its interaction with hBMECs^20^. Being a principle components of OMVs^24,25^ and encoded by *mafA* gene located on the *maf* meningococcal pathogenicity island-like region wherein majority of the genes are related to virulence factors such as toxins, adhesins or invasins^22^, the probability of the involvement of MafA in host-pathogen crosstalk and neuroinvasion is high. Hitherto, very few studies have reported transcriptomic data on the host cells challenged with *Neisseria*^43,44^, whereas such data are not available from the hBMECs challenged with neisserial antigen. Both earlier studies^43,44^ have used microarray, which is not a sufficient technology to draw complete picture of cellular transcriptome. In this report, we attempted to reveal complete transcriptomes induced by NM or MafA using high-throughput RNA-seq technology followed by comprehensive dissection of the set of the genes according to various biological processes. The important signaling events in each biological process are discussed below.

Bacterial adhesion on non-phagocytic cells (like epithelial and endothelial cells) causes dramatic cytoskeletal rearrangement^11,45–48^. Cytoskeletal rearrangement begins with clustering of membrane receptors and structural proteins beneath the site of bacterial attachment, which leads to the formation of cortical plaques^11,47,48^. Subsequently, members of the family of G-protein coupled receptors (e.g. β-2 adrenergic receptor) recruit β arrestin, which mediates several cellular processes needed for internalization of NM^48,49^. In the present study, transcripts belonging to the G-protein coupled receptor signaling pathway - adrenomedullin (Adm), Adm2 and arrestin domain containing 3 (ARRDC3) were upregulated in hBMECs exposed to NM. Although not identical, two genes of G-protein coupled receptor signaling pathway – adhesion G protein coupled receptor (ADGR), and ADGRL were upregulated in MafA exposed hBMECs. Subsequent signaling events in the formation of cortical plaques include activation of Src kinase^50^, GTPases Rho, Cdc42^11^ and Rac1, recruitment of cortactin mediated by phosphoinositide-3-kinase (PI3K)^9^ and activation of MAP kinase. In the present study, gene transcripts of Src family tyrosin kinase – LYN, Rho GTPase 1 and 3 were upregulated in both hBMECs exposed to NM or MafA. Furthermore, CD44 that initiates cell signaling cascade involving activation of Rac1 and cytoskeleton linker protein ezrin (leading to tyrosine phosphorylation of cellular proteins followed by cytoskeletal rearrangement^51^) was evoked in cells challenged with NM (logFC 2.7) as well as MafA (logFC 2.1). The cortical plaque induces regulation and translocation of proteins present at intercellular junctions *viz*., VE-cadherin, claudin and catenin^12^ to the site of bacterial adhesion and helps in the formation of microvilli like membrane protrusions that protect bacterial colonies from the blood flow shear stress. Concomitant recruitment of VE-cadherin (*CDH5*) into cortical plaques depletes the stock of junctional proteins resulting in paracellular openings and crossing of the bacteria through the BBB^12^. Expression of VE-cadherin was evoked moderately in hBMECs, while, the major adhesion molecules VCAM-1, ICAM-1 and E-selectin present in the microvilli like membrane protrusions were highly upregulated. It is noteworthy to realize that, signaling pathways activated by NM during its interaction with endothelial cells are similar to those promoted by leukocyte during their attachment on hBMECs^52^. These signaling pathways are presented in supplementary dataset S5 online^53–55^ and the similarity of the events observed after NM (slide 1) and MafA (slide 2) challenges are highlighted. Upregulated VE-cadherin, claudin 1, claudin 14 and adhesion molecules in hBMECs exposed to both MafA and whole bacteria denotes that alike whole bacterial cell, MafA is capable of inducing events required for the formation of membrane like protrusions.

The protrusions surrounding adhered *Neisseria* lead to the endocytosis. Significant upregulation of the genes taking part in the formation of endosomes (e.g. SCARB2, SGIP, etc.) and antigen processing (cathepsin S, CTSS) indicates that the process of endocytosis was evoked. Note, however, that the gene expression of RAB5C that promotes docking of vesicles to the correct acceptor compartment was downregulated only in hBMECs challenged with MafA. It was shown previously that Opc of *Neisseria* binds with integrin receptors at the apical surface of hBMECs leading to receptor-mediated endocytosis^17^. The expression profile of DEGs having a role in the endocytosis is similar between hBMECs induced with NM and MafA (Fig. 5), which may indicate the role of MafA in endocytosis alike Opc. Another member of opacity-associated protein family - Opa binds to the CEACAM1 receptor, which evokes expression of members of CEACAM family and mediates bacterial uptake, cellular transcytosis and release of bacteria at the basolateral surface^56^. Gene transcript of CEACAM1 was upregulated in hBMECs exposed to MafA and NM in our study. It would be interesting to know whether NM exploits MafA besides opacity proteins to interact with CEACAM proteins of brain endothelial cells for endocytosis.

*N. meningitidis* causes detachment of BMECs from the matrix (typical feature of anoikis) along with complete dissociation of membrane occludins and dislodgement of tight junctions^36^. It was further reported that the metalloproteinases released by infected hBMECs aid this process^36^. Detachment of the challenged endothelial cells were noticed in our study (data not shown), and metalloproteinases such as ADAM8 and ADAM17 were evoked. Dislodgement of the endothelial cells may initiate apoptosis through caspase-independent manner^36^, while, it is known that meningococcal infection induces apoptotic signals^57^. In case of MafA exposed cells several genes directly associated with apoptotic signaling were induced in the same extent (e.g. tumor necrosis factor ligand superfamily member 10 (*TNFSF10*)^58^ and TNF^59,60^) as that of NM. Moreover, genes coding death receptors such as *TNFSF1B* and Fas cell surface death receptor were upregulated. It is well known that activation of both receptors triggers formation of the death-inducing signaling complex which stimulates recruitment of FADD (Fas-associated death domain) and activation of caspase-8 resulting in apoptosis of the cells^61–63^. Members of the Bcl-2 protein family, notable candidates in the cytochrome c mediated signaling of the cell death^64,65^, those having proapoptotic effect were upregulated in our study e.g. PUMA (*BBC3* NM logFC 2.31, MafA logFC 1.54), NOXA (*PMAIP1* NM logFC 2.16, MafA logFC 2.00), and Bim (*BCL2L11* NM logFC 1.49, MafA logFC 1.35). Involvement of these genes in the apoptotic pathway and their augmentation caused by NM and MafA are depicted in supplementary dataset S5 online (slides 3 and 4)^53–55^. It is important to note that, simultaneous expression of pro – and anti apoptotic genes or concurrent up and downregulation of proapoptic genes may occur in the cells in response to the infection. In the cells undergoing NM infection, concurrent upregulation of pro apoptotic *Bak* and *Bad* genes, and downregulation of *Bax* and death-associated protein 3 were reported earlier^37^. In our study, expression of the Bcl-2-modifying factor which is an apoptotic activator^66^ was augmented (*BMF* logFC 1.89), whereas expression of *Bad* was downregulated (*BAD* logFC – 1.57) in hBMECs challenged with NM. An expression of the *Bad* remained unchanged in hBMECs induced with MafA.

hBMECs have been reported to express Toll-like receptors (TLRs) 1, 2, 3, 4 and 6, which induce proinflammatory cytokines and chemokines^5,6^. hBMECs exposed to both NM and MafA were observed to have 5 and 2 fold overexpression of TLR2 and TLR 3 genes, respectively. Further on, transcripts of adaptor molecule - MyD88 and subsequent downstream genes *viz*. p105, IκBα, NF-κB, and AP1 transcription factor subunit were observed to have enhanced expression. Previously, neisserial porin B was demonstrated to bind TLR2 and activate the downstream cascade in MyD88 dependent manner^8^. The upregulated transcripts of TLR2 and MyD88 in our study supports previous finding that NM stimulates TLR2 - MyD88 in hBMECs^8^, and indicate that MafA could be an additional ligand besides porin B. Upregulated transcripts of TLR3 in hBMECs exposed to NM and MafA is an unique observation of this study.

Even though endothelial cells do not fall in the category of typical immune cells, they still produce substantial amount of inflammatory responses through cytokines^13^. Exposure of hBMECs to NM or MafA resulted in upregulation of transcripts belonging to proinflammatory cytokines *viz*. TNFα, IL1β, IL-6, IL-8 and RANTES. Among those, pro-inflammatory regulators - IL1β and TNFα were shown to enhance production of cell adhesion molecules including ICAM-1 and VCAM-1^13^. Saukkonen *et al*.^67^ have shown that pneumococci cause profound induction of the inflammatory molecules (TNF, IL1α, IL1β) leading to the increase in the BBB permeability. Thus, we believe that highly expressed TNFα (6 to 8 fold), IL1β (6 to 6.9 fold), IL-6, IL-8 and RANTES in hBMECs denote profound inflammatory response in hBMECs exposed to NM and MafA that can be beneficial for bacterial adhesion and may compromise BBB permeability. Pro-inflammatory cytokines are also known to induce expression of MHC I in BMECs^68^. Genes encoding MHC class I A, B and F were upregulated in infected and MafA exposed hBMECs (Fig. 7). It is noteworthy to observe significant overexpression (4 to 8 fold) of genes encoding C-X-C motif chemokine ligands, C-C motif chemokine ligands and a C-X3-C motif chemokine ligand in hBMECs exposed to both NM and MafA. These chemokines primarily attracts neutrophils and monocytes^16,69^. Rats infected with *Klebsiella pneumoniae* causing meningitis had shown overexpressed CXCL2 chemokines in cerebrospinal fluids^70^. Increased expression of chemokines in our study may suggest that MafA is able to induce regulation of chemokines in hBMECs and may initiate neutrophil infiltration.

One of the major advantages of the RNA-seq over microarray is that it can identify DEGs belonging to non coding RNA and pseudogenes. Several species of non protein coding RNAs were found in this study. Of them, the expression of antisense RNA transcripts was found altered in challenged cells (NM 10 upregulated, 2 downregulated; MafA 6 upregulated, 5 downregulated). Antisense RNAs are complementary to mRNA, which can attenuate the mRNA transcription. Other classes of non-coding RNAs observed in our RNA-seq include - lincRNA, processed pseudogenes and transcribed processed pseudogenes (Supplementary dataset S4 online). Expressed lincRNAs have shown to be involved in cancer, regulation of chromatin structure, scaffolding of proteins and can sequester activity of the protein, mRNA and microRNA^71^. Although pseudogenes were disregarded to have any function and termed “junk genes”, recent studies have shown that they are expressed to regulate several protein coding genes^72^. We have observed several processed and unprocessed pseudogenes in hBMECs exposed to both NM and MafA (Supplementary dataset S4 online). Pseudogenes are reported to repress the expression of the corresponding mRNA^72–74^. It is interesting to note that, number of pseudogenes expressed in hBMECs was higher in MafA compared to NM infected cells, and the majority of them were downregulated except guanylate binding protein 1 pseudogene 1. Guanylate binding proteins are categorized into GTPases participating in cell-autonomous immunity against bacterial pathogens and are induced by interferons^75^. Another important class of non protein coding RNAs are mitochondrial RNAs (mt-RNA) that synthesize proteins through mitochondrial own translational machinery consisting of mt-tRNA (22 genes) and mt-rRNA (2 genes)^76^. Four mt-tRNA transcripts (MT-TY logFC −4.11, MT-TP logFC −3.05, MT-TL1 logFC −2.57, MT-TF logFC −2.05) were significantly downregulated only in MafA challenged cells. The downregulation of mt-tRNA can result in insufficient production of proteins involved in respiratory chain^76^. On the other hand, both mt-rRNAs (MT-RNR1 logFC 2.34 and MT-RNR2 logFC 2.36) in MafA challenged cells were upregulated. To our knowledge, the specific function of the non coding RNA transcripts in the bacterial infection is scanty, and further studies are necessary to understand their role in host-pathogen interactions.

In summary, this work has attempted to map comprehensive picture of the ongoing biological processes in hBMECs in response to *N. meningitidis* and MafA. This is the first report in which RNA-seq was used to reveal transcriptome in the hBMECs evoked by NM. Several biological processes were revealed in transcriptome data, however, we have attempted to present and discuss only major events those may help *Neisseria* during the translocation cross BBB. On the basis of similar profiles of DEGs (in BMECs challenged with NM and MafA) belonging to cell surface modification leading to membrane protrusion (VCAM-1, ICAM-1, E-selectin, VE-cadherin, claudin 1, claudin 14) and receptor mediated endocytosis (probably through CEACAM) it is speculated that *N. meningitidis* might use MafA to interact with hBMECs. The genes belonging to death signaling (TNFSF10, BBC3, BCL2L11, PMAIP1, BMF, TRADD, FAS and TNFRSF10B) observed in our study be dependent on changes occurring in extracellular matrix organization due to NM infection. hBMECs seemed to produce substantial amount of inflammatory responses through cytokines to resist neisserial infection and translocation across the BBB. Nevertheless, profound induction of the inflammatory molecules (TNFα, IL1β, IL-6, IL-8 and RANTES) may increase the permeability of BBB benefiting invading pathogen. MafA seems to be important adhesin of NM with potential to provoke expression of DEGs associated with cytokine response, anoikis and apoptosis. The work first time highlights ability of MafA to induce biological processes in the endothelial cells which may help *Neisseria* to invade BBB.

## Materials and Methods

### Culture of human brain microvascularendothelial cells

Human BMECs (hBMEC/D3 cell line), were obtained from Merck/Millipore (Prague, Czech Republic). Cells were cultured as previously described in our previous publication^77^, while further details are in the supplementary information Method S1 online.

### Culture of N. meningitidis

*N. meningitidis* serogroup B (isolate M1/03) was kindly provided by The University Hospital Olomouc, Czech Republic. The isolate was cultured from the blood of the patient suffering from neisserial meningitis. The isolate was characterized by phenotyping (biochemical tests) and genotyping (multilocus sequence typing). *mafA* gene amplified and sequenced (Genbank MK940370) from this isolate had 99.8% homology with mafA of the neuroinvasive *Neisseria meningitidis* MC58 serogroup B (GenBank AE002098), whereas the amino acid sequences similarity with MafA of MC58 was 100%. This isolate was used in the study mainly because of its low passage, neuroinvasiveness, high virulance and known clinical symptoms. *N. meningitidis* was cultured as described in our previous publication^20^. In brief, very low passaged (P02) isolate was inoculated on Thayer Martin agar (Becton-Dickinson, USA) and a single isolated colony was transferred into 50 mL of Brain Heart Infusion Broth (BHI) enriched with 10 mM MgCl_2_. Culture was grown at 37 °C and 5% CO_2_ until OD_600_ = 0.6 (mid-exponential phase).

### Synthesis of recombinant MafA

MafA was overexpressed in *E. coli* expression system. In brief, gene fragment encoding MafA was amplified by PCR from genomic DNA of *N. meningitidis* serogroup B (isolate M1/03). Detailed information on primer, amplicon length and restriction enzymes used are presented in supplementary information Table S2 online. Details of the digestion of amplified PCR product, ligation into a pQE-30-mCherry-STOP plasmid, transformation and selection of clones are presented in supplementary information Method S2 online. Insertion of the encoding gene was confirmed by sequencing with vector specific primers UA Insertom F and R, (Supplementary information Table S2 online). Steps in the protein induction and purification are presented in details supplementary information Methods S2 and S3 online. The purity of the recombinant protein was assessed by SDS-PAGE, while molecular mass was measured by MALDI-TOF MS (details are presented in supplementary information Method S4 online). Protein concentration was measured with Bradford method.

### Challenge of hBMECs

hBMECs cultured in 6-well plates were incubated either with live NM (MOI 0.5, bacterial cells were washed with minimal essential medium, Sigma) or recombinant MafA (approximately 1 nMol, 27 μg/well) or non-related ligand of *Streptococcus pneumoniae* (as non-related protein control, approximately 1 nMol/well) or just the culture medium (non-induced control) for 6 hours at 37 °C in the presence of 5% CO_2_. Please note that recombinant non-related ligand of *Streptococcus pneumoniae* used here was produced using same conditions described for recombinant MafA (unpublished data). This non-related ligand is one of the endothelial cell binding proteins of *S. pneumoniae* described in our previous publication^77^.

After incubation, mRNA from hBMECs was isolated using RNeasy Mini Kit (Qiagen, Germany) according to manufacturer’s instructions. Of note, DNaseI (Qiagen) treatment was essentially incorporated during RNA isolation. The RNA concentration was measured on nanodrop (Thermo scientific) and it was stored at −80 °C in aliquots. Integrity of RNA was monitored using a capillary electrophoresis (Fragment analyzer, Advanced Analytical Technologies, Inc, USA).

### Preparation of the library

250 ng of RNA was reverse transcribed with oligodT primers for synthesis of the first strand cDNA using QuantSeq. 3′ mRNA-Seq Library Prep Kit (Lexogen, Austria). All steps described below were performed exactly as per the manufacturer’s instructions. RNA template was removed with RNA removal solution (RS buffer, Lexogen) and the second strand was synthesized using random hexamer primer that contains Illumina-compatible linker sequences at its 5′ end. The double stranded DNA libraries were purified using magnetic beads provided in the kit. Each library was amplified by PCR using unique single indexing i7 primers to add complete adapter sequence required for cluster generation and to generate sufficient DNA for sequencing and quality control. The number of cycles in PCR for each library was determined using PCR Add-on kit for Illumina (Lexogen). Cycles used for library amplification were as follows: hBMECs induced with NM *−* 20 cycles, hBMECs induced with MafA - 20 cycles, hBMECs induced with non-related ligand *-* 19 cycles, non-induced cells − 17 cycles. Amplified libraries were purified using magnetic beads supplied in the kit. Quality of the libraries and length of the fragments were checked on fragment analyzer.

### NGS Sequencing and data analysis

Libraries were sequenced on Illumina NextSeq, single-end 75 bp, to a minimal depth 8 million reads per sample. Fastq files were processed and aligned to reference genome (*Homo sapiens* GRCh38) using STAR aligner (STAR V 2.5.2b, (https://github.com/alexdobin/STAR/releases/tag/2.5.2b). The pre-processing includes adaptor trimming and removal of initial 10 bases (recommended for QuantSeq as these bases are random-priming sites). Reads were counted in STAR V 2.5.2b. To perform differential gene expression analysis edgeR an open source R package version 3.12 was used (https://bioconductor.org/packages/release/bioc/html/edgeR.html). The low read count with less than 3 CPM (count per million) were filtered out using the filterByExp function of edgeR package. The identification of differentially expressed gene (DEGs) was accomplished by using glmTreat and glmQLFit (quasi-likelihood, QL) functions of edgeR in R package considering log fold change (logFC) values beyond ± 1.2 and FDR less than 0.05.

The logical relation of DEGs between the challenged hBMECs (MafA or NM; MafA or non-related ligand) was calculated using Excel (MS office) and Venn diagrams were constructed. To group DEGs into GO biological processes, Reactome server was used (https://reactome.org/) and to construct heat maps, Heatmapper server was used (http://www.heatmapper.ca/expression/). Signaling pathways related to the translocation across the BBB (cell adhesion molecules, leukocyte transendothelial migration, regulation of actin cytoskeleton, ECM-receptor interaction, focal adhesion, PI3K-AKT signaling pathway and apoptosis) were downloaded from KEGG server (https://www.genome.jp/kegg/) and the DEGs involved in the pathways were manually highlighted.

### Validation of DEGs by qRT-PCR

RNA was reverse transcribed into cDNA using random hexamers (Thermo Scientific). Briefly, 1 μg of RNA and 100 pMol of random hexamers were mixed and incubated 5 minutes at 65 °C. Subsequently, 4 μL of 5x reaction buffer, 2 μL dNTP (10 mM), 1 μL RevertAid reverse transcriptase (200 U) (Thermo Fisher Scientific, USA) and 0.5 μL RiboLock RNase inhibitor (20U) (Thermo Fisher Scientific, USA) were added. The reaction mixture was incubated 10 minutes at 25 °C, 1 hour at 42 °C followed by 70 °C for 10 minutes.

A set of DEGs significantly up and downregulated in RNA-seq (6 upregulated and 4 downregulated) were selected for qRT-PCR. Primers used in qRT-PCR were designed using Geneious Pro software (Biomatters, USA) and are presented in supplementary information Table S2 online. Reaction mix of qRT-PCR composed of 6 ng of cDNA, 1x qPCR GreenMaster with highROX (Jena Bioscience, Germany), gene-specific primers (10 pMol each) and RNase free water up to total volume of 20 μL. Each reaction was performed in triplicates. Amplification cycles were as follows: 95 °C – 10 minutes, 40 × [95 °C – 15 s., 50–60 °C – 30 s (annealing temperature varied according to the primers used), 72 °C for 30 s (signal capture)], melting curve 60 °C to 95 °C – 0.3% temperature increment/s. (StepOnePlus, Thermo Fisher Scientific, USA). The gene expression (ΔΔCt) was normalized to β–2-microglobulin (house-keeping gene) as described before^78^. ΔΔCt values were converted to logFC (http://www.endmemo.com/algebra/log2.php). The expression values for DEGs obtained from RNA-seq and qRT-PCR were correlated with Pearson correlation coefficients PCCs using on-line server - https://www.socscistatistics.com/tests/pearson/Default2.aspx

### Construction of *mafA* knock-out mutant (Δ*mafA* NM), induction of hBMECs with Δ*mafA* NM and analysis of gene expression

Δ*mafA* NM was constructed by homologous crossover, which replaced *mafA* gene with *bla* gene (which encodes beta-lactamase). Detailed information on the construction of the cassette used for the homologous crossover is described in supplementary information Method S5 online. The cassette was constructed with overlapping extension PCR (OE-PCR). Schematic presentation of OE-PCR is in supplementary information Fig. S6 online. Transformation of NM with cassette and selection of the Δ*mafA* mutants is described in supplementary information Method S6 online.

hBMECs were cultured maintaining the same conditions as are described in supplementary information Method S1 online. Cells were challenged either with Δ*mafA* NM or wild type M1/03 NM (NM wt) for 6 hours exactly as described above. Non-induced cells were used as a negative control. The experiment was performed in biological triplicates. After incubation, RNA was isolated, reverse transcribed into cDNA and used for qRT-PCR as described above. ΔΔCt and logFC were calculated as mentioned above. Statistical difference between gene expressions (logFC) observed in wt and Δ*mafA* NM was calculated with unpaired *t* test (https://www.graphpad.com/quickcalcs/ttest1).

## Supplementary information

Supplementary dataset 1

Supplementary dataset 2

Supplementary dataset 3

Supplementary dataset 4

supplementary dataset 5

Supplementary information

## Data Availability

The datasets generated during and/or analysed during the current study are available from the corresponding author on reasonable request.
