# Supplementary material for: Transcriptome analysis of human brain microvascular endothelial cells response to Neisseria meningitidis and its antigen MafA using RNA-seq
Source: Sci Rep. 2019 Dec 10;9:18763. doi: 10.1038/s41598-019-55409-y (PMC6904618; doi:10.1038/s41598-019-55409-y)
Supplement: Supplementary file 5 — supplementary dataset 5 [file 41598_2019_55409_MOESM5_ESM.pdf]

Transcriptome analysis of human brain microvascular endothelial cells response to *Neisseria meningitidis* and its antigen MafA using RNA-seq

Evelína Káňová[1], Zuzana Tkáčová[1], Katarína Bhide[1], Amod Kulkarni[1], Irene Jiménez-Munguía[1], Patrícia Mertinková[1], Monika Drážovská[1], Punit Tyagi[1], Mangesh Bhide[1,2*]

The schematic view of the events in the leukocyte and its resemblance with expressed genes in the hBMECs challenged with NM (Slide 1) and MafA (Slide 2).

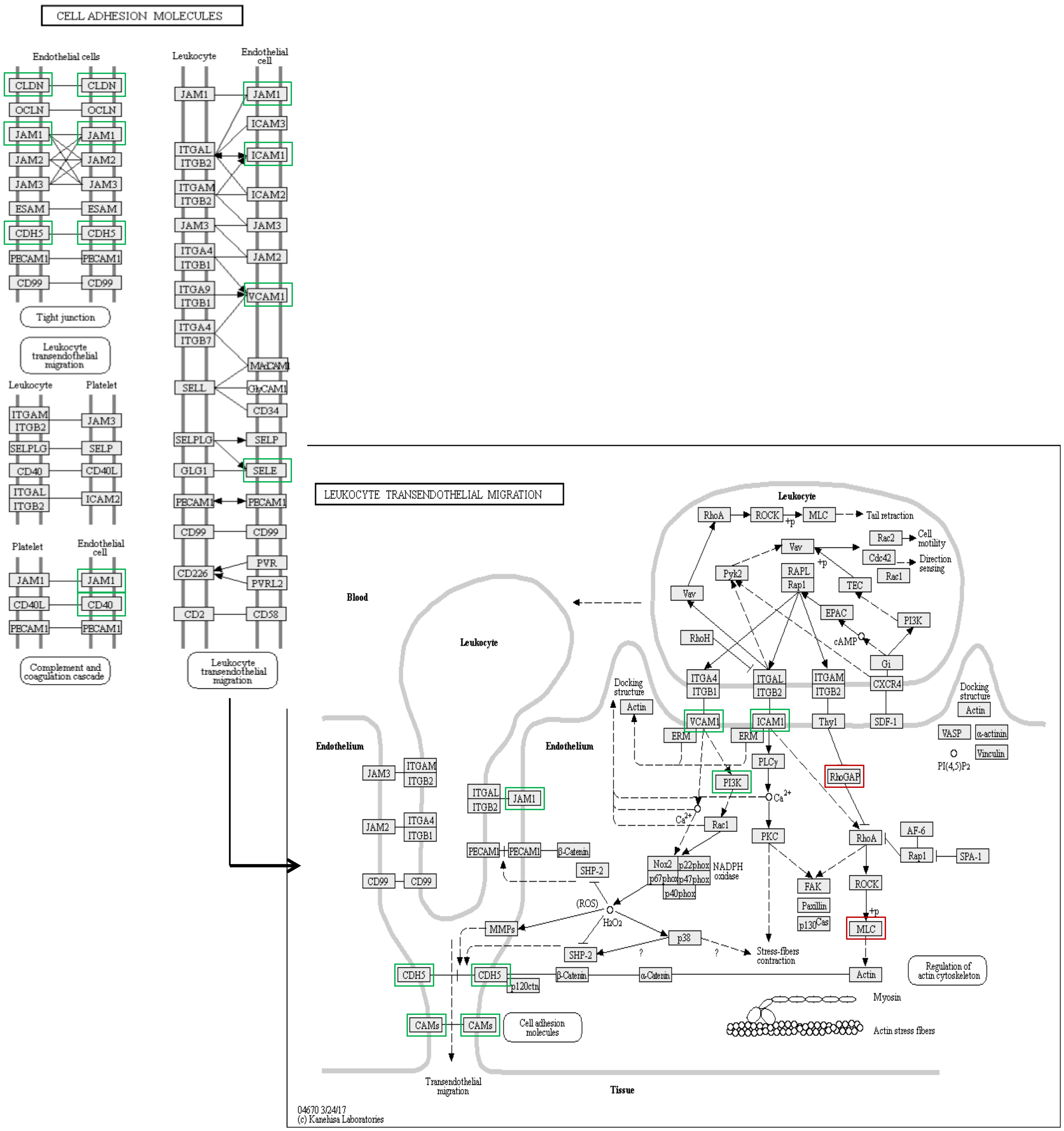

Green boxes -upregulated genes; red boxes- downregulated genes. transendothelial migration

Pathways are downloaded from the "KEGG" (Kyoto Encyclopedia of Genes and Genomes). Please see citations 53-55 in the main article.

The schematic view of the events in the leukocyte transendothelial migration and its resemblance with expressed genes in the hBMECs challenged with NM (Slide 1) and MafA (Slide 2).

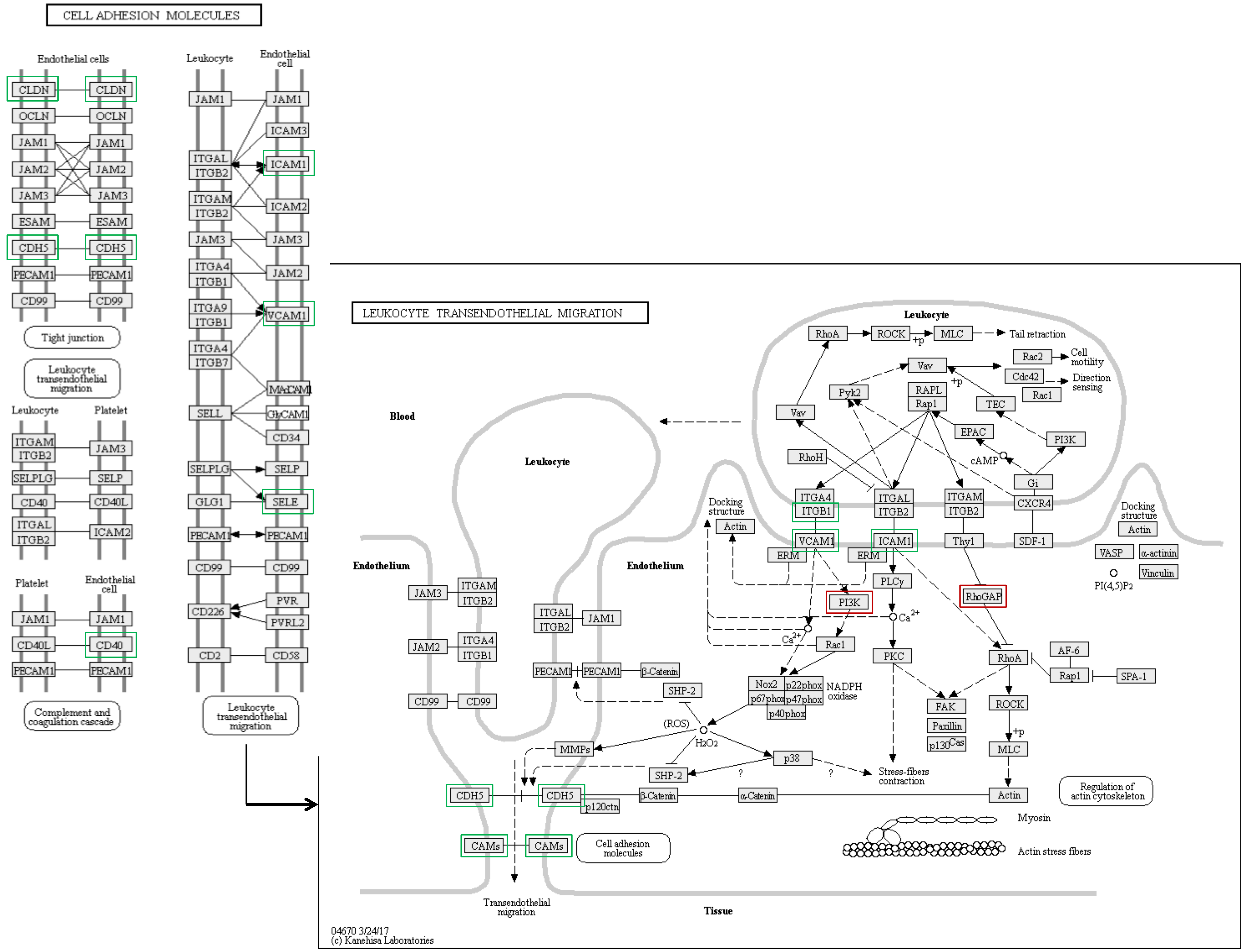

Green boxes -upregulated genes; red boxes- downregulated genes.

Pathways are downloaded from the "KEGG" (Kyoto Encyclopedia of Genes and Genomes). Please see citations 53-55 in the main article.

Schematic view of the events in the focal adhesion leading to the activation of PI3K signaling pathway and apoptosis in the hBMECs induced with NM (Slide 3) and MafA (Slide 4)

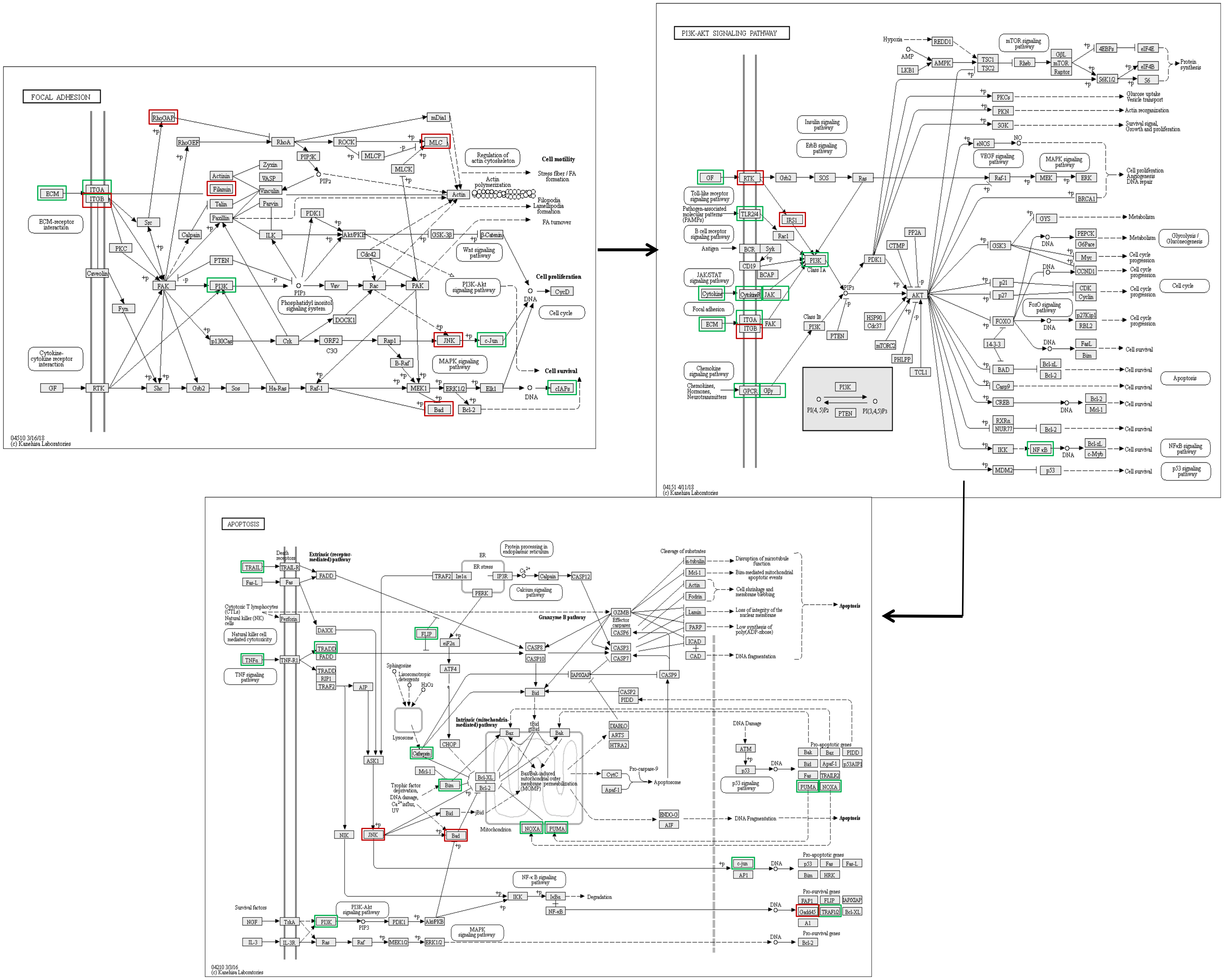

Green boxes -upregulated genes; red boxes- downregulated genes.

Pathways are downloaded from the "KEGG" (Kyoto Encyclopedia of Genes and Genomes). Please see citations 53-55 in the main article.

Schematic view of the events in the focal adhesion leading to the activation of PI3K signaling pathway and apoptosis in the hBMECs induced with NM (Slide 3) and MafA (Slide 4)

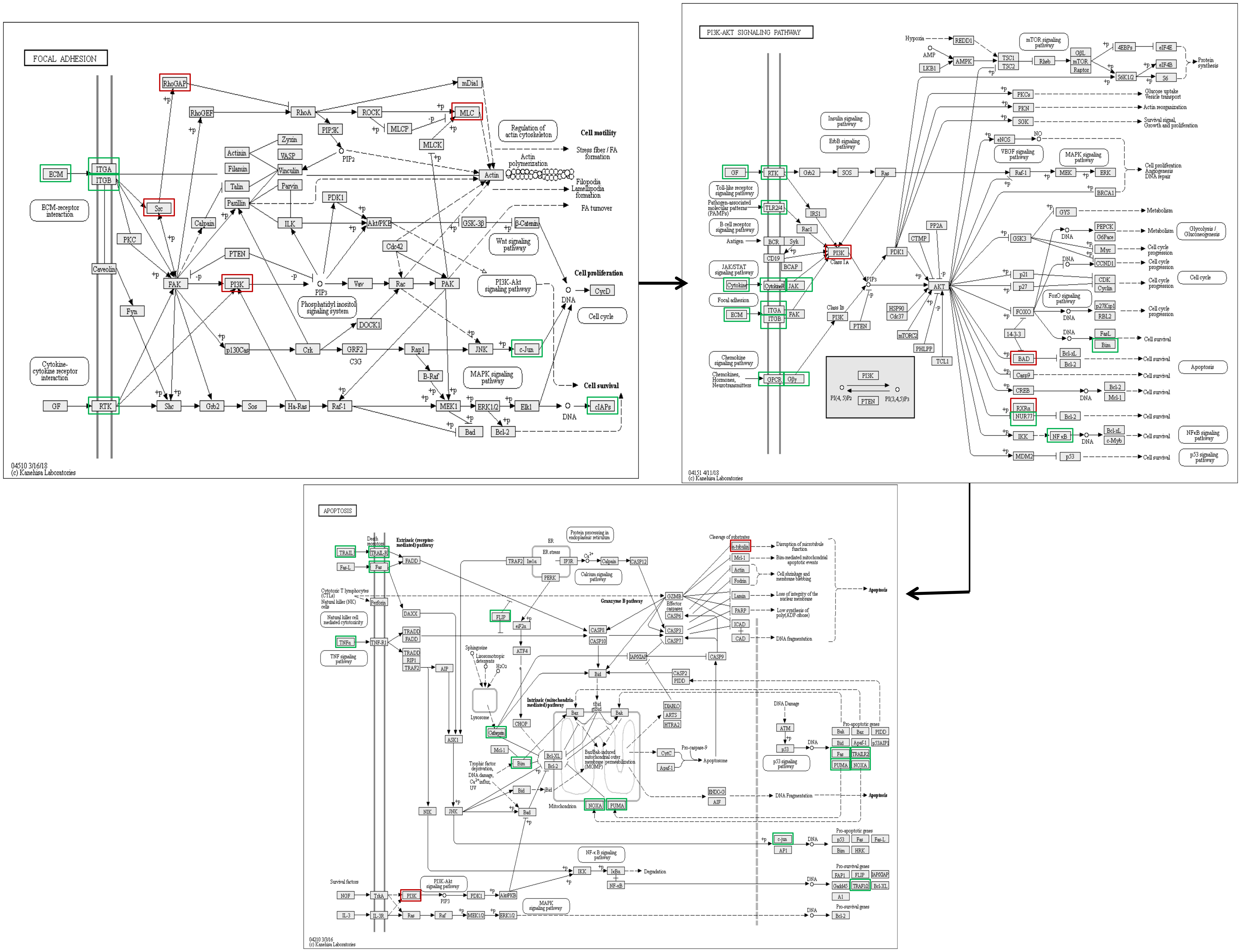

Green boxes -upregulated genes; red boxes- downregulated genes.

Pathways are downloaded from the “KEGG” (Kyoto Encyclopedia of Genes and Genomes). Please see citations 53-55 in the main article.
