# Supplementary material for: Transcriptome analysis of human brain microvascular endothelial cells response to Neisseria meningitidis and its antigen MafA using RNA-seq
Source: Sci Rep. 2019 Dec 10;9:18763. doi: 10.1038/s41598-019-55409-y (PMC6904618; doi:10.1038/s41598-019-55409-y)
Supplement: Supplementary file 6 — Supplementary information [file 41598_2019_55409_MOESM6_ESM.pdf]

Transcriptome analysis of human brain microvascular endothelial cells response to *Neisseria meningitidis* and its antigen MafA using RNA-seq

Evelína Káňová[1], Zuzana Tkáčová[1], Katarína Bhide[1], Amod Kulkarni[1], Irene Jiménez-Munguía[1], Patrícia Mertinková[1], Monika Drážovská[1], Punit Tyagi[1], Mangesh Bhide[1,2*]

SUPPLEMENTARY FIGURES

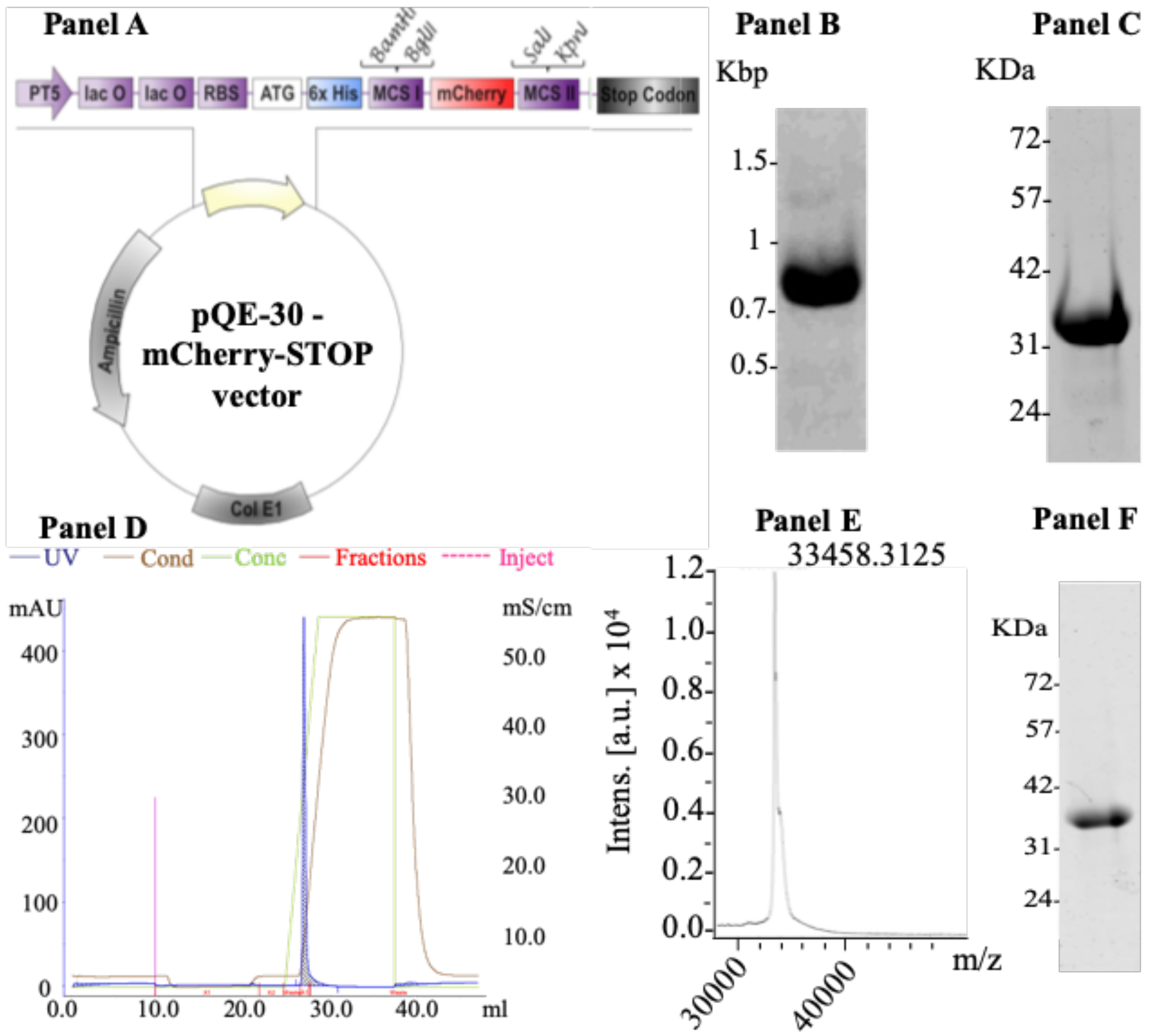

Supplementary Fig. S1. Steps in the production of recombinant MafA.

Panel A. pQE-30-mCherry-STOP plasmid (4880bp). Col E1 origin of replication, ampicillin resistance gene, PT5 T5 promoter, lac O lac operator, RBS ribosome binding site, ATG Start codon, 6xHis tag sequence, MCSI/MCSII multiple cloning sites, mCherry- red fluorescent protein that serve as stuffer, Stop codon. Backbone pQE-30 was obtained from Qiagen, Germany.
Panel B. Production of recombinant form of MafA. Amplicon of the gene coding fragment of MafA resolved on agarose gel;
Panel C. Recombinant protein separated with nickel affinity chromatography, resolved SDS-PAGE and stained with silver staining;
Panel D. Cation exchange – chromatograph of MafA purification.
Panel E. Molecular mass of the recombinant MafA confirmed with MALDI-TOF/MS.
Panel F. Purified MafA after cation exchange and endotoxin removal. Protein resolved on SDS-PAGE and stained with silver staining.

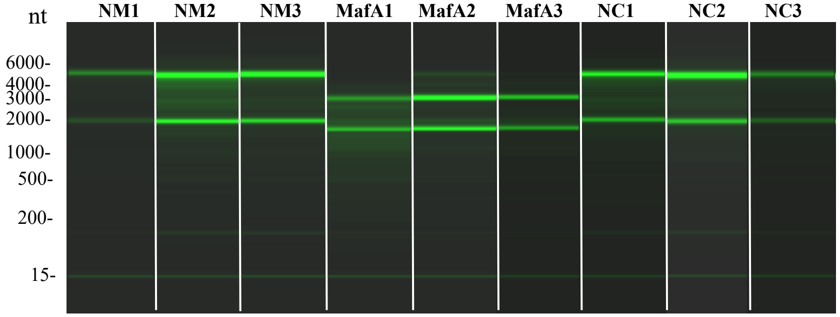

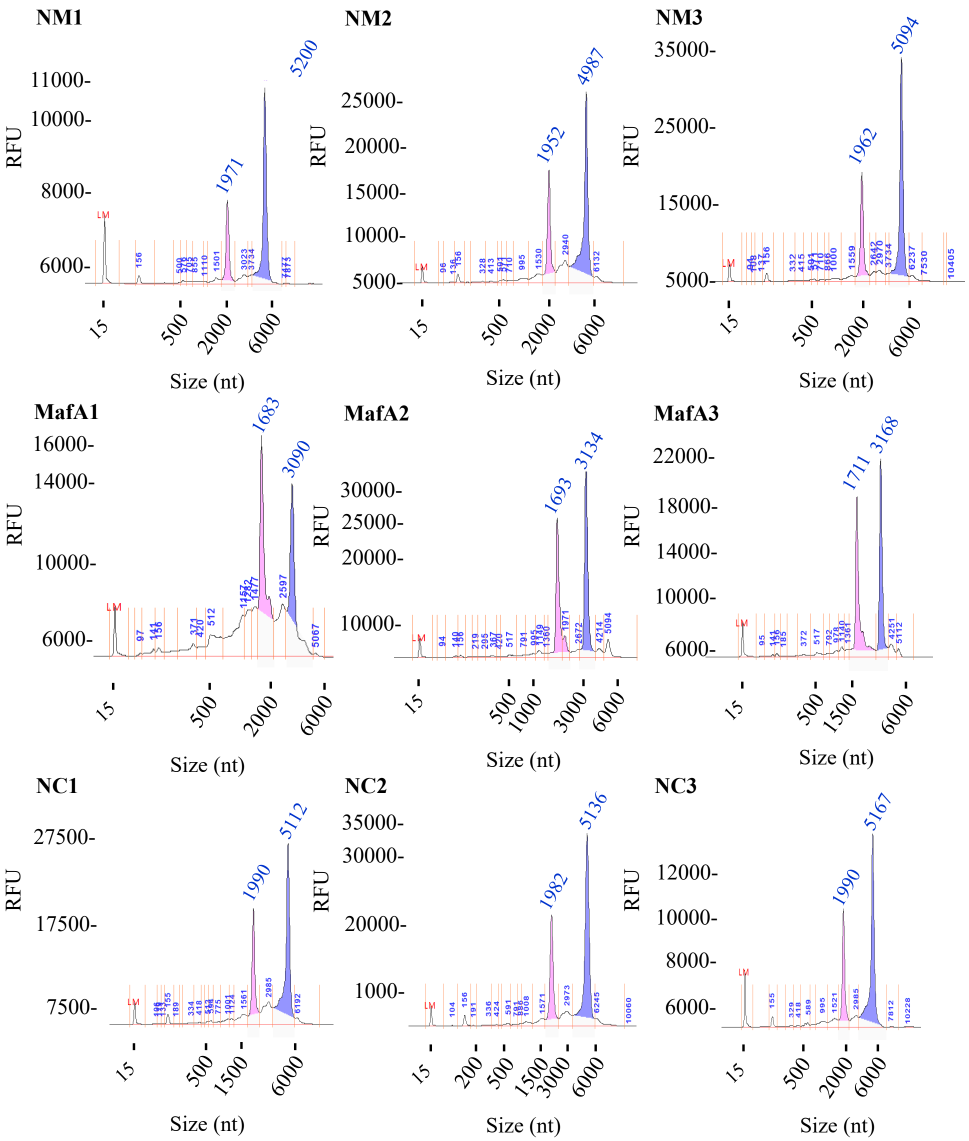

Supplementary Fig. S2. Upper figure (Electrophoresis) - assessment of integrity of RNA isolated from hBMECs incibated with NM (NM1,2,3), MafA (MafA1, 2, 3,) or without any protein (negative control, NC1, 2, 3).

Graphs on the right (NM1 to NC3) - graphical representation of peaks of RNA derived from electrophoresis. RFU – relative fluorescence unit

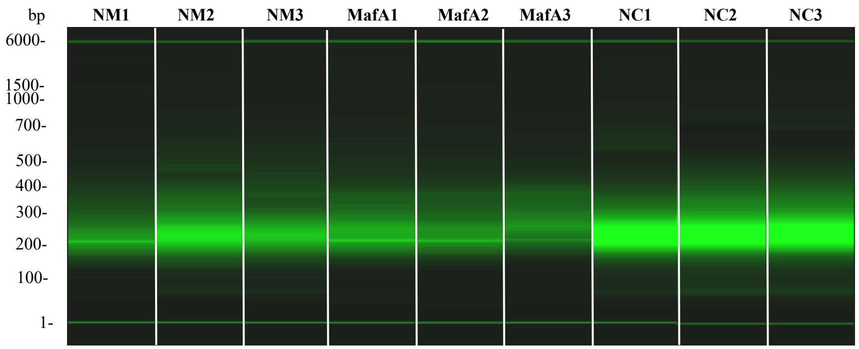

Supplementary Fig. S3. Upper figure (Electrophoresis) - Quality control of libraries prepared with QuantSeq 3‘ mRNA kit used for sequencing. hBMECs induced with NM (NM1,2,3) or MafA (MafA1, 2, 3) or without any protein (negative control, NC1, 2, 3). Note that the ideal fragment size should be between 150-300 bp.

Graphs on the right (NM1 to NC3) - graphical representation of the peaks of DNA fragments derived from electrophoresis. RFU – relative fluorescence unit

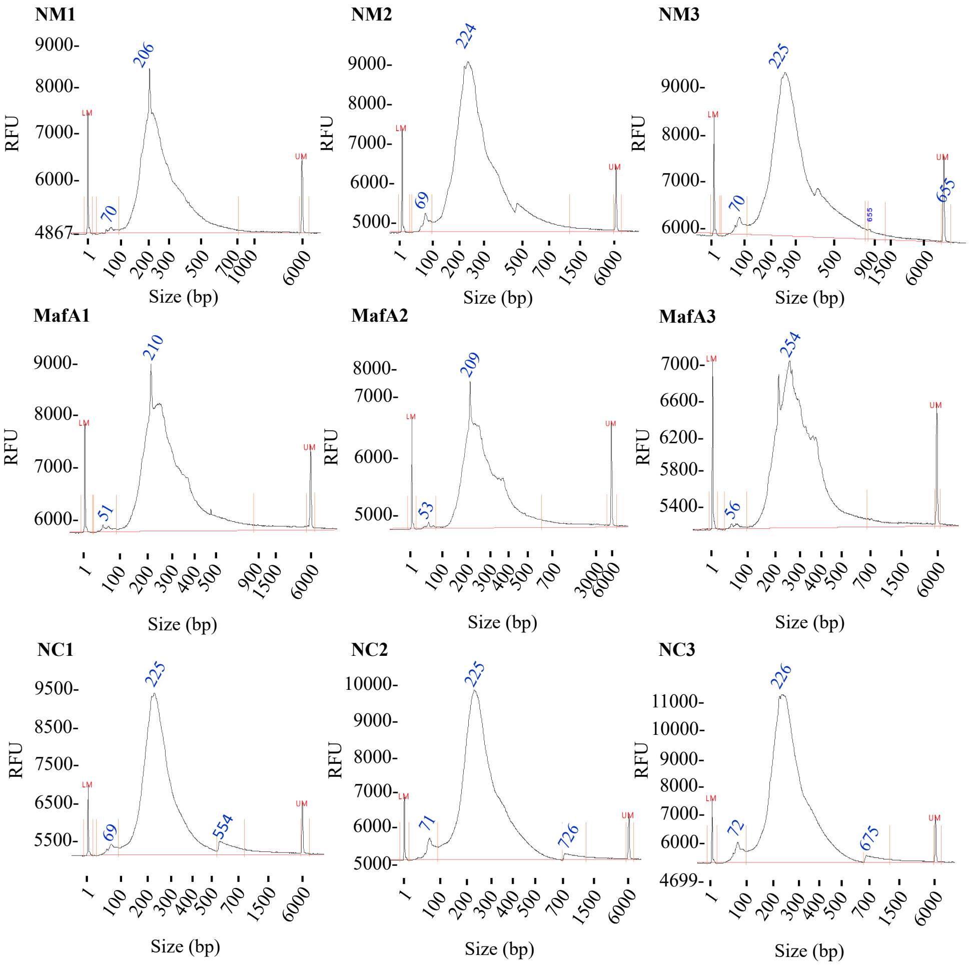

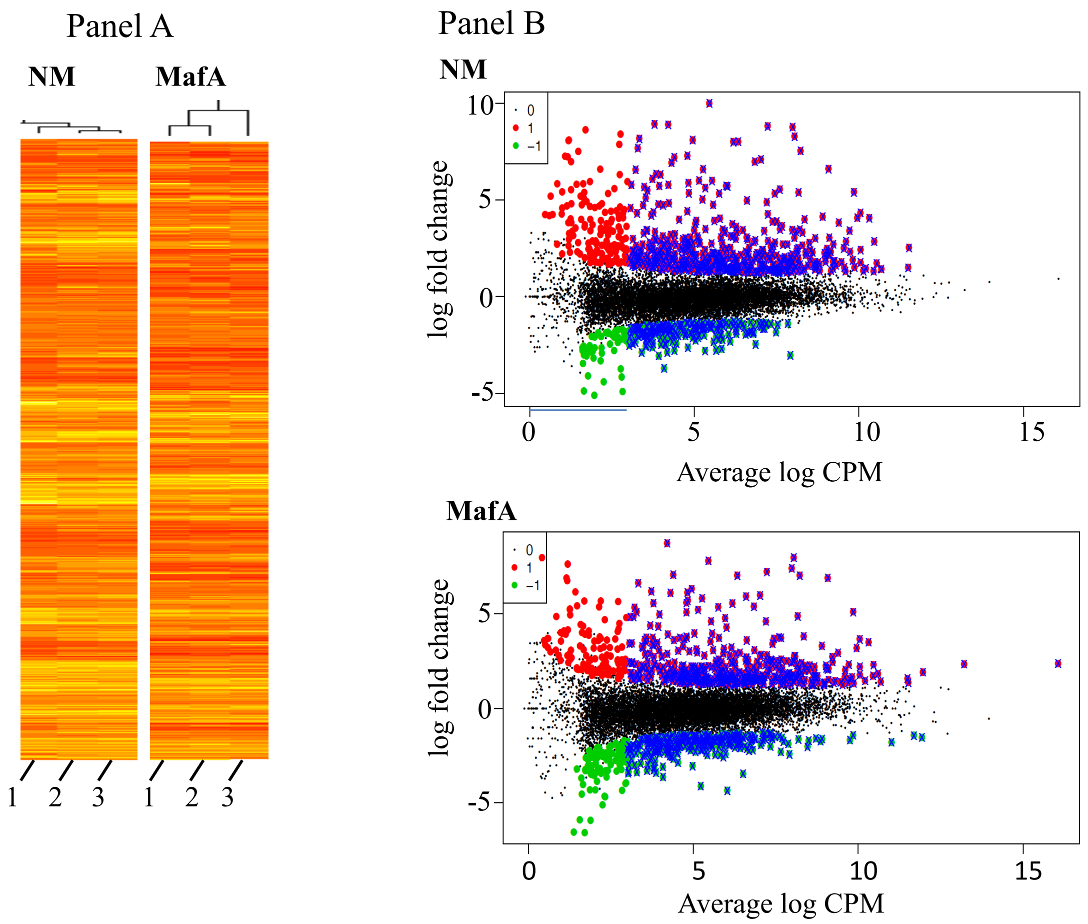

Supplementary Fig. S4. Panel A. Heat map generated from sequence reads (1,2,3 represents triplicates). **Panel B.** A scatter plot showing genes (each dot presents a single gene). Log fold change with +/- 1.2 was used to select DEGs. All genes which are within this range are presented with black dots (non significant). A cutoff of 3 for average log CPM was set. All the genes more than this cutoff were selected as DEGs and presented with blue cross.

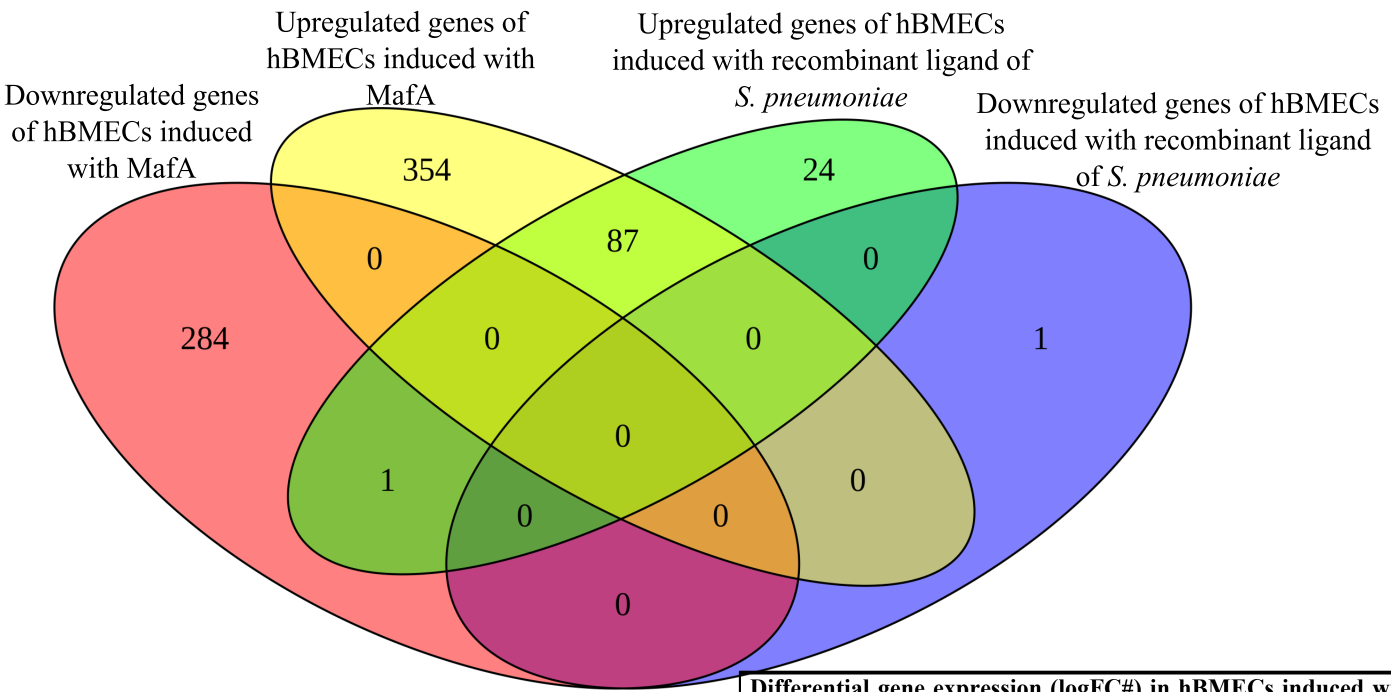

Supplementary Fig. S5. Venn diagram – Comparison between DEGs evoked by recombinant MafA and recombinant ligand of *S. pneumoniae* (non-related control). Please note that, MafA evoked 726 DEGs, whereas with non-related protein evoked genes were only 113.

Only 87 genes were common between two treatments. Table on the righthand side: Differential gene expression in the hBMECs induced with MafA and non-related protein. Please note that only selected genes are presented here. Whole list of DEGs with logFC is in Supplementary dataset 2.

Differential gene expression (logFC#) in hBMECs induced with recombinant MafA and non-related protein *(recombinant ligand of S. pneumoniae)*

gene	hBMECs induced with MafA	hBMECs induced with recombinant ligand of *S. pneumoniae*
ICAM-1	3.97	2.21
VCAM	7.03	3.12
IL-6	5.7	2.43
IL-8 (CXCL8)	7.97	3.6
CXCL10	7.22	3.16
CXCL11	5.55	3.59
TIMP4	-2.45	No differential expression*
NPR3	-2.06	No differential expression
TSC22D3	-2.43	No differential expression
SLC16A12	-3.64	No differential expression

* Differential expression calculated against non-induced control

\# LogFC values are from RNA-seq analysis

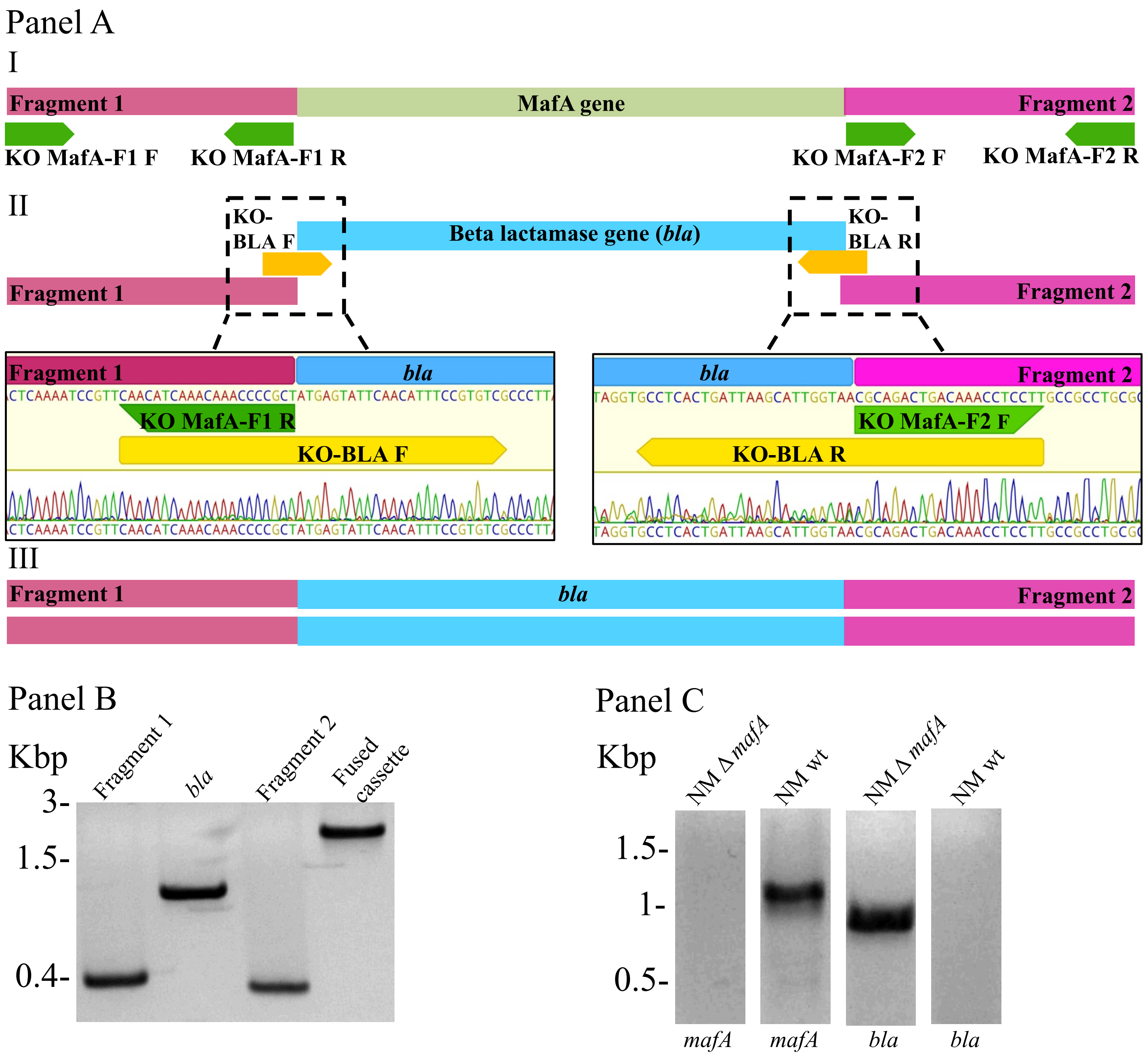

Panel A
I
Fragment 1
MafA gene
Fragment 2
KO MafA-F1 F
KO MafA-F1 R
KO MafA-F2 F
KO MafA-F2 R
II
KO-BLA F
Beta lactamase gene (*bla*)
KO-BLA R
Fragment 1
Fragment 2
Fragment 1
bla
KO MafA-F1 R
KO-BLA F
bla
Fragment 2
KO MafA-F2 F
KO-BLA R
III
Fragment 1
bla
Fragment 2
Panel B
Kbp
Fragment 1
bla
Fragment 2
Fused cassette
3-
1.5-
0.4-
Panel C
Kbp
NM Δ *mafA*
NM wt
NM Δ *mafA*
NM wt
1.5-
1-
0.5-
mafA
mafA
bla
bla

Supplementary Fig. S6. Steps involved in deletion of *mafA* gene

Panel A- Schematic representation of construction of fused cassette used to knock-out *mafA* gene.

363 bp upstream (fragment 1) and 318 bp downstream (fragment 2) sequences to *mafA* gene were amplified with primers depicted in I.

bla gene encoding beta-lactamase was amplified from pLEXY I-ble3 using primers shown in II.

(sequences of primers are in **Supplementary Table S2).**

Fragment 1, bla and fragment 2 were fused with overlap extension PCR (OE-PCR). As depicted in II, KO-BLA F and KO-BLA R primers (used to amplify *bla*) have 21 bp overlaps to fragment 1 and fragment 2, respectively. These overlaps ensure fusion of three fragments during the first round of OE-PCR in direction – *5‘-fragment 1-bla-fragment 2 – 3‘*

Fused cassette (as depicted in III) was amplified using PCR-product from first step of OE-PCR and primers KO-MafA-F1-F and KO-MafA-F2-R.

Fused cassette was sequenced to corroborate proper fusion. Chromatograms of the fused regions are presented in II.

Panel B- Agarose gel depicting amplicons of the fragment 1, *bla* gene, fragment 2 and full fused cassette after second round of OE-PCR.

Panel C- Confirmation of knock-out *mafA* was performed by PCR. DNA from NM clones from BHI-carbenicillin agar was isolated and tested for absence of *mafA* and presence of *bla*. Results of only one clone is presented here. Absence of *mafA* and presence of *bla* in NM Δ *mafA* are presented. As a control, wild type (NM wt, M1/03) was used, in which *mafA* is present and *bla* is absent.

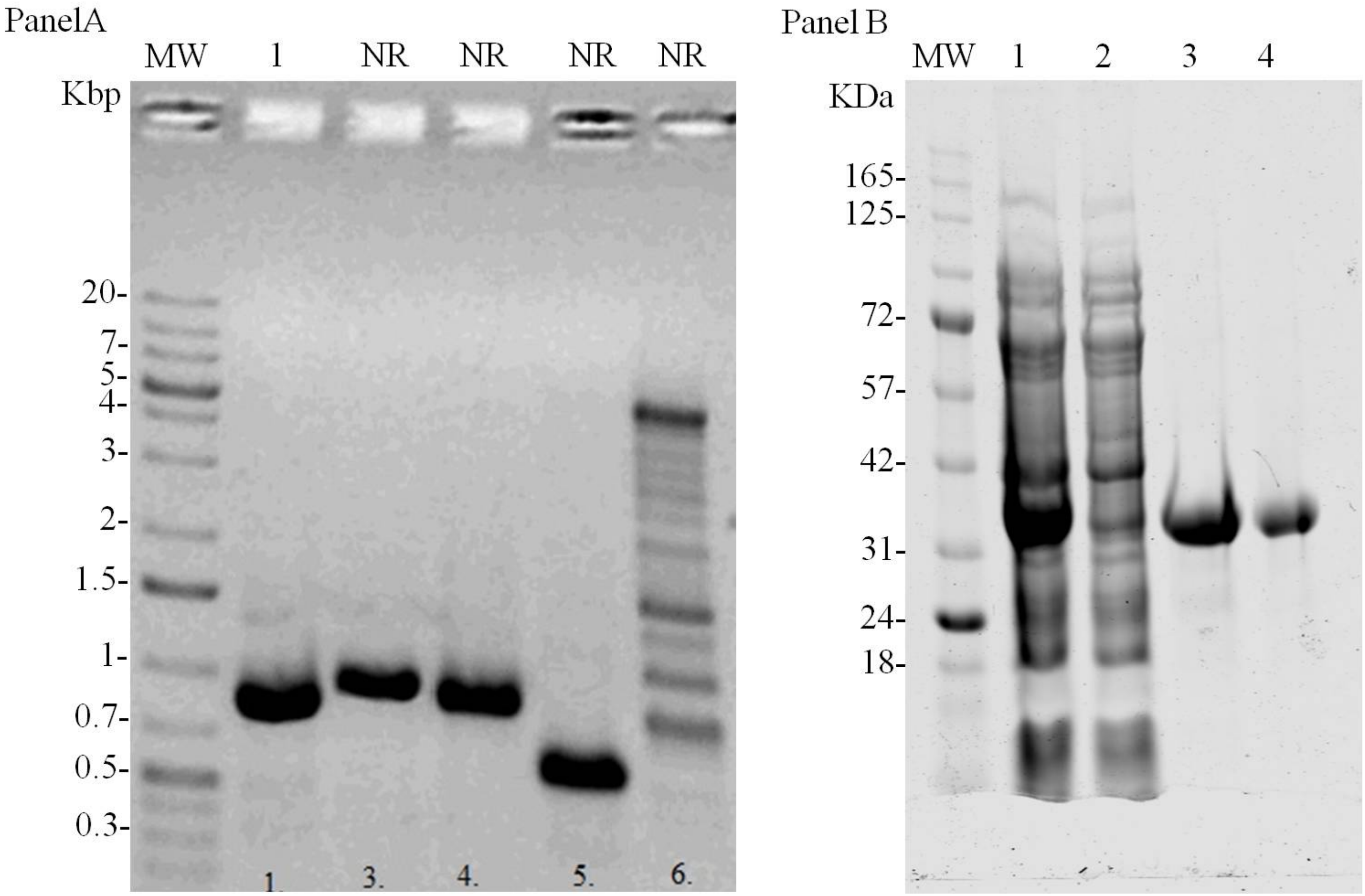

Supplementary Fig. S7. Original figure used to make the Supplementary Fig. S1. Panel A- production of recombinant form of MafA. Amplicon of the gene coding fragment of MafA resolved on agarose gel (Lane 1); NR – PCR products of coding fragments of other genes not included in this study. Panel B- recombinant protein MafA separated on SDS-PAGE after Nickel affinity chromatography (Lane 3); whole cell lysate of *E. coli* containing MafA (Lane 1); Flow-through from nickel affinity chromatography (Lane 2); Purified recombinant MafA-2nd elution (after polishing with cation exchange and endotoxin removal - Lane 4).

Panel A

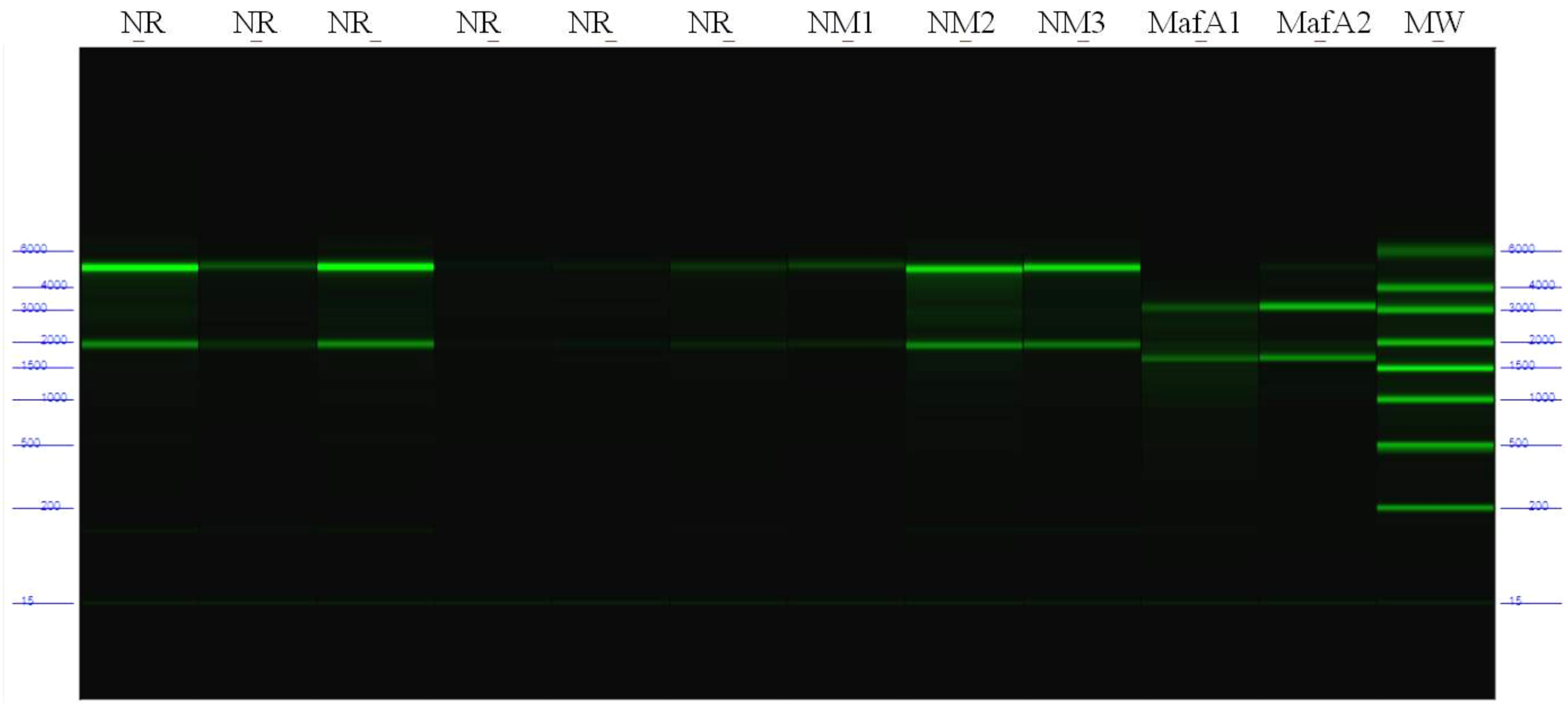

Supplementary Fig. S8. Original figures used to make Supplementary Fig. S2. Panels present assessment of integrity of RNA isolated from hBMECs challenged with *N. meningitidis* (NM1,2,3 in Panel A), MafA (MafA1,2,3 in Panels A and B) or without any protein-negative control (NC 1,2,3 in Panels B and C).
NR - nonrelevant sampels for this study; B - blanc.

Panel B

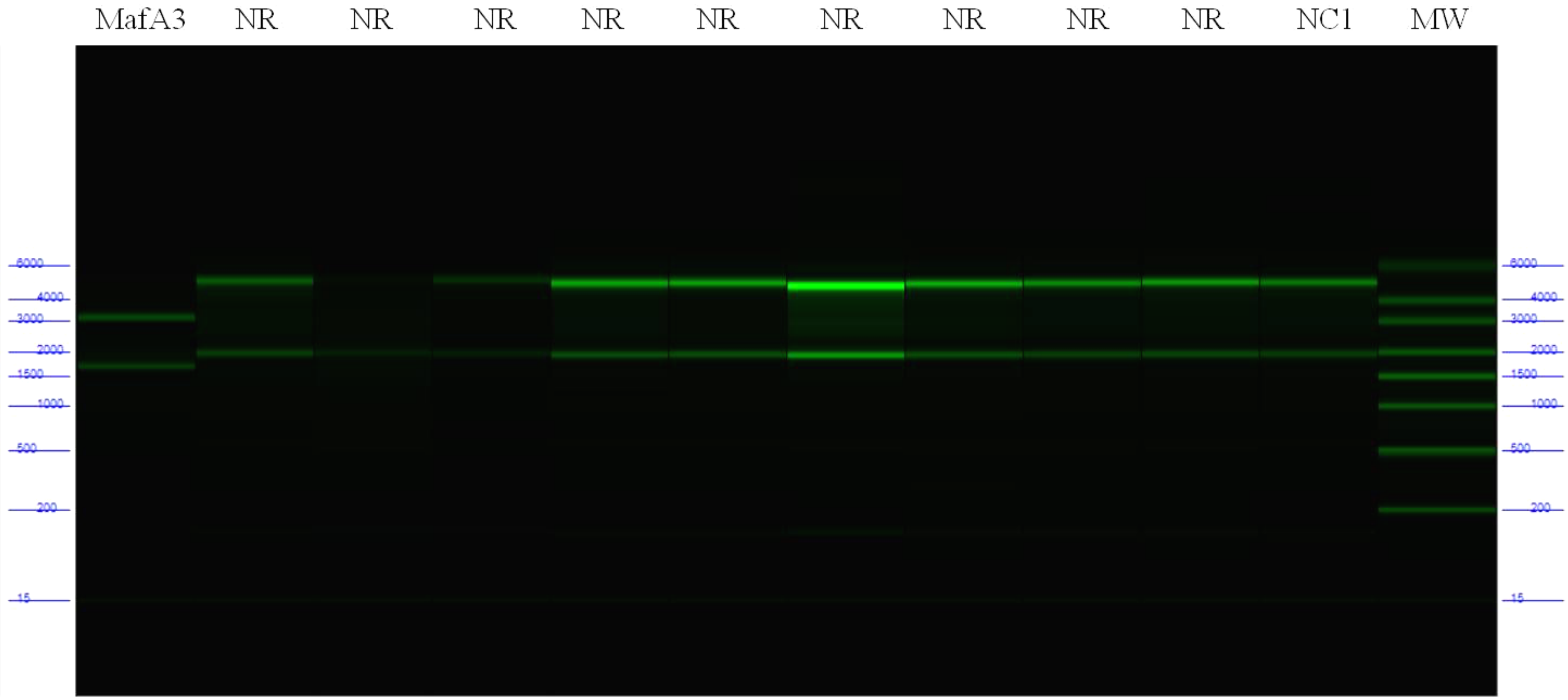

Supplementary Fig. S8. Original figures used to make Supplementary Fig. S2. Panels present assessment of integrity of RNA isolated from hBMECs challenged with *N. meningitidis* (NM1,2,3 in Panel A), MafA (MafA1,2,3 in Panels A and B) or without any protein-negative control (NC 1,2,3 in Panels B and C).
NR - nonrelevant sampels for this study; B - blanc.

Panel C

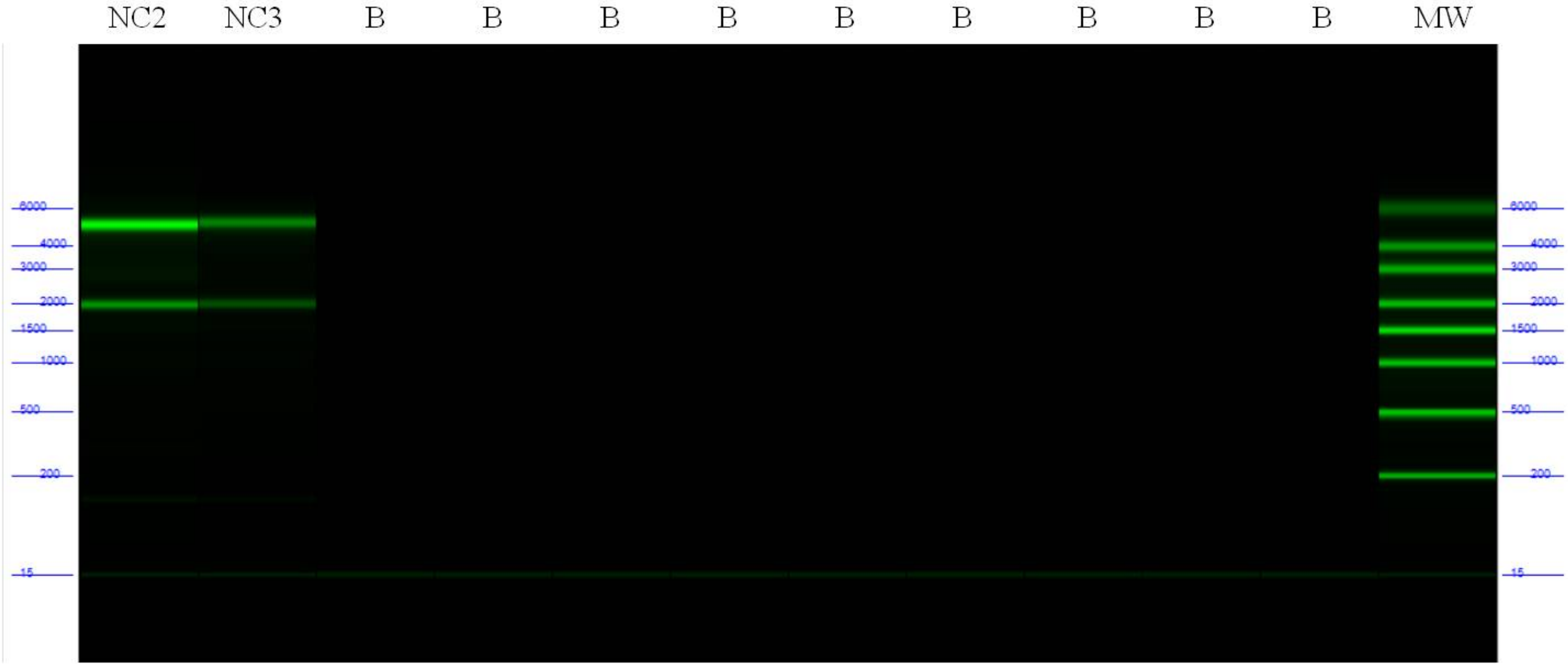

Supplementary Fig. S8. Original figures used to make Supplementary Fig. S2. Panels present assessment of integrity of RNA isolated from hBMECs challenged with *N. meningitidis* (NM1,2,3 in Panel A), MafA (MafA1,2,3 in Panels A and B) or without any protein-negative control (NC 1,2,3 in Panels B and C).
NR - nonrelevant sampels for this study; B - blanc.

Panel A

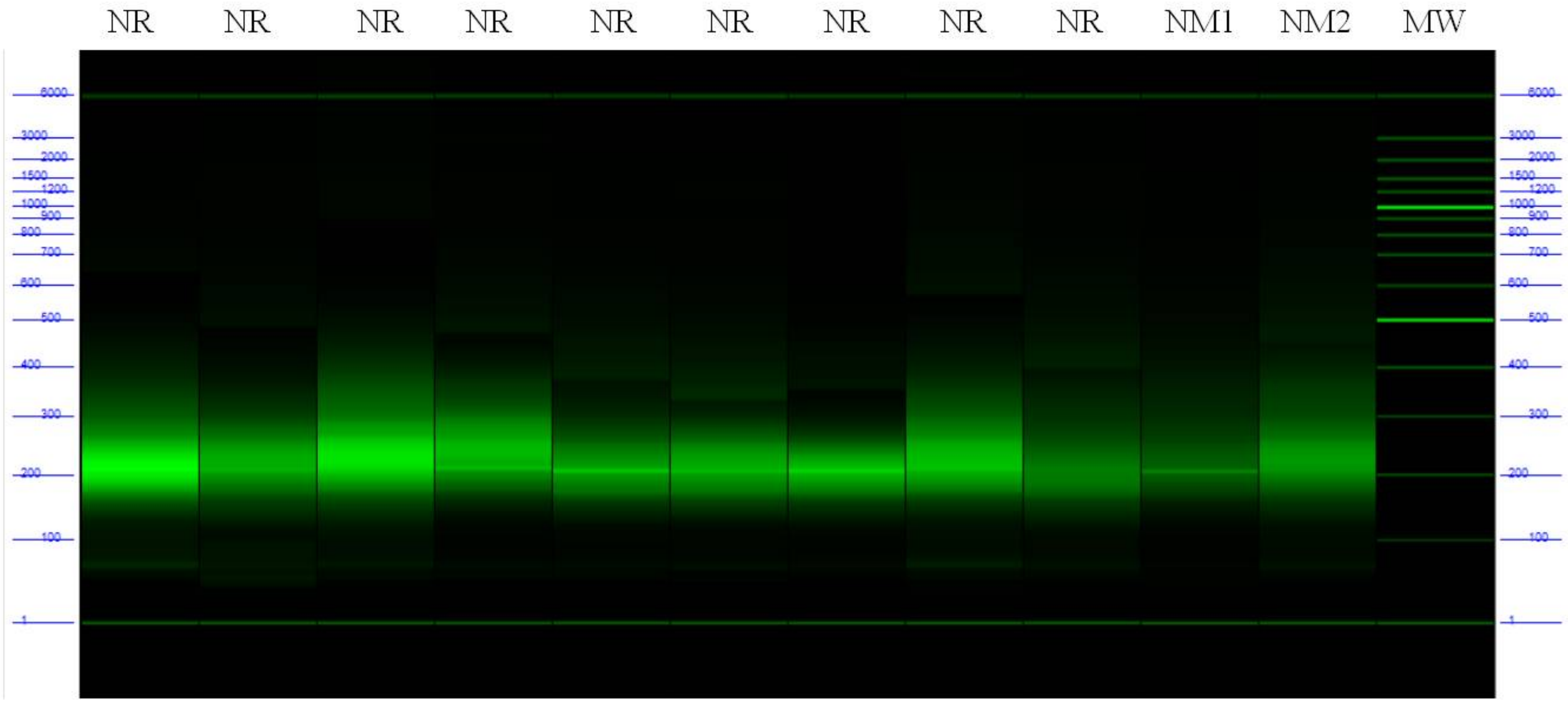

Supplementary Fig. S9. Original figure for making the Supplementary Fig. S3. Panels present quality control of libraries prepared with QuantSeq 3‘ mRNA kit used for sequencing. hBMECs challenged with *N. meningitidis* (NM1,2,3 in Panels A and B), MafA (MafA1,2,3 in Panel B) or without any protein-negative control (NC 1,2,3 in Panels B and C). NR - nonrelevant samples for this study.

Panel B

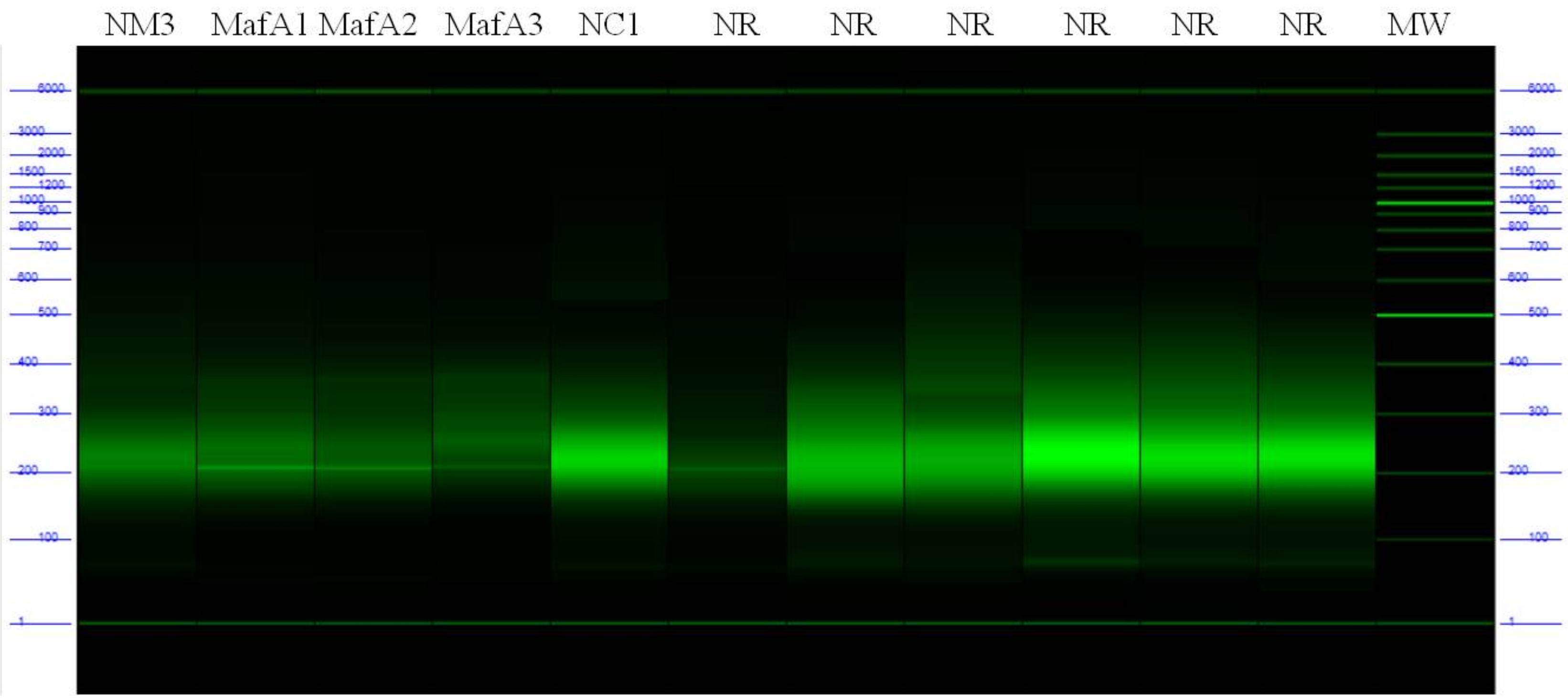

Supplementary Fig. S9. Original figure for making the Supplementary Fig. S3. Panels present quality control of libraries prepared with QuantSeq 3' mRNA kit used for sequencing. hBMECs challenged with *N. meningitidis* (NM1,2,3 in Panels A and B), MafA (MafA1,2,3 in Panel B) or without any protein-negative control (NC 1,2,3 in Panels B and C). NR - nonrelevant samples for this study.

Panel C

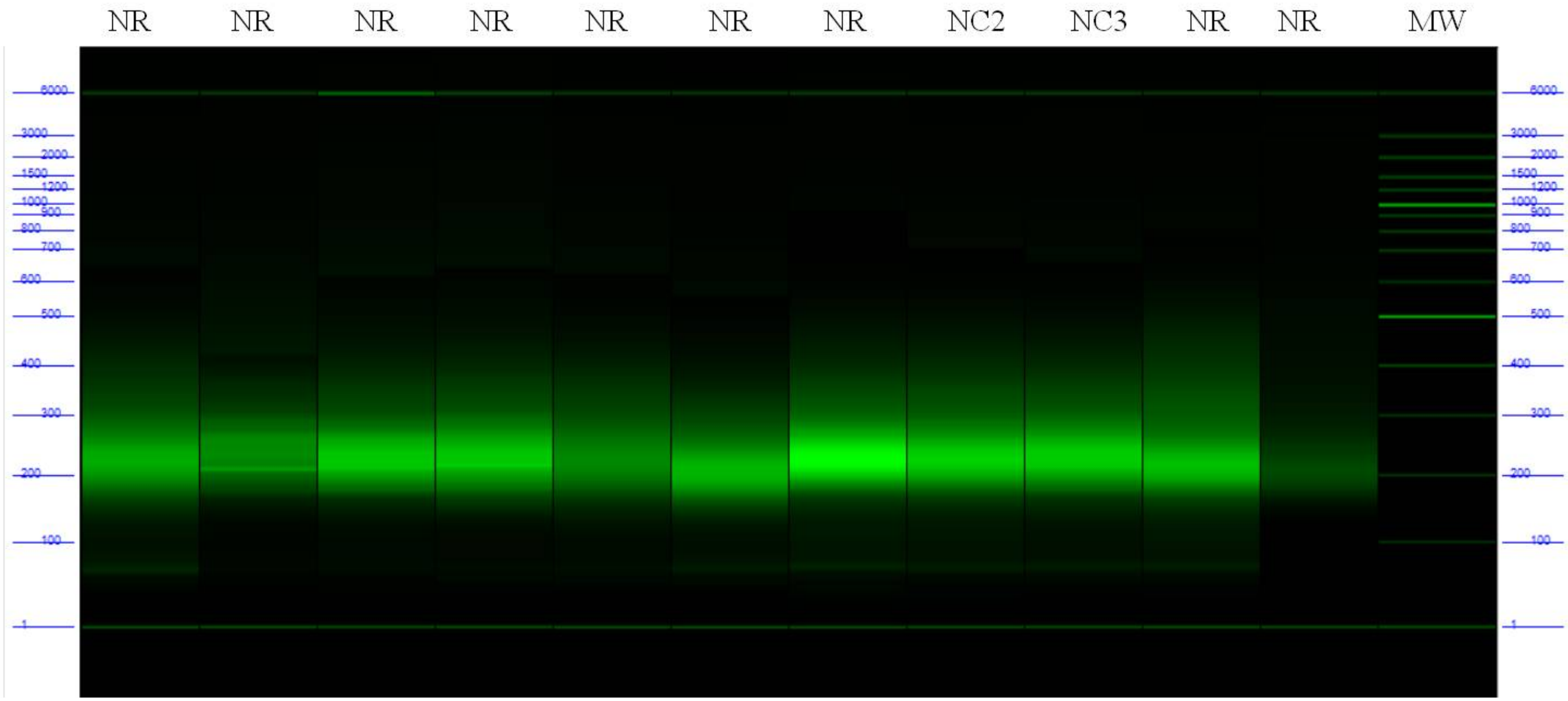

Supplementary Fig. S9. Original figure for making the Supplementary Fig. S3. Panels present quality control of libraries prepared with QuantSeq 3‘ mRNA kit used for sequencing. hBMECs challenged with *N. meningitidis* (NM1,2,3 in Panels A and B), MafA (MafA1,2,3 in Panel B) or without any protein-negative control (NC 1,2,3 in Panels B and C). NR - nonrelevant samples for this study.

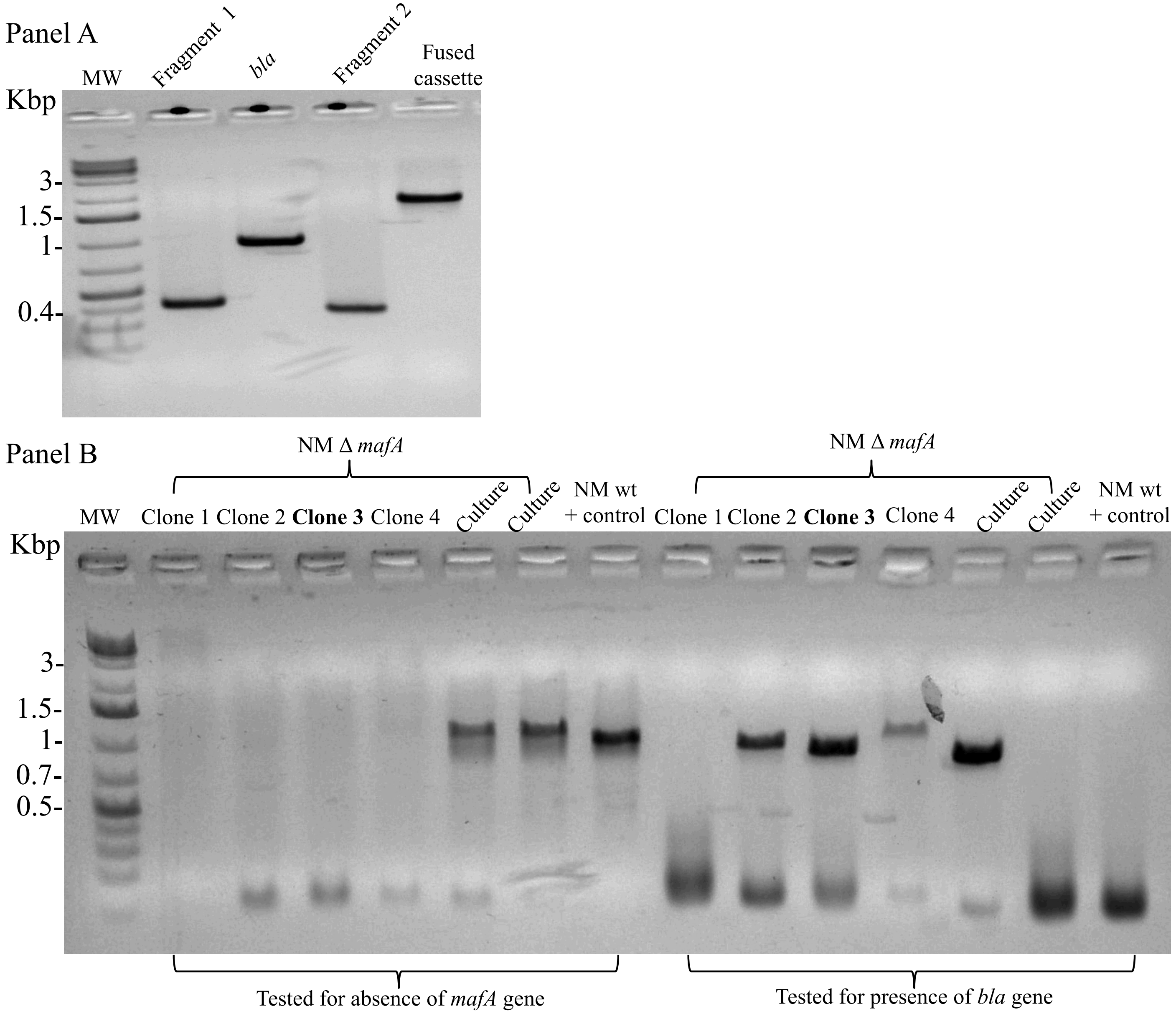

Panel A
MW
Fragment 1
bla
Fragment 2
Fused cassette
Kbp
3-
1.5-
1-
0.4-
Panel B
NM Δ mafA
NM Δ mafA
MW
Clone 1
Clone 2
Clone 3
Clone 4
Culture
Culture
NM wt + control
Clone 1
Clone 2
Clone 3
Clone 4
Culture
Culture
NM wt + control
Kbp
3-
1.5-
1-
0.7-
0.5-
Tested for absence of mafA gene
Tested for presence of bla gene

Supplementary Fig. S10. Original figures used to make Supplementary Fig. S6.
Panel A- Agarose gel depicting amplicons of the fragment 1, *bla* gene, fragment 2 and full fused cassette after second round of OE-PCR.
Panel B- Confirmation of knock-out *mafA* was performed by PCR. DNA from NM clones from BHI-carbenicillin agar (clones 1- 4) and from BHI-carbenicillin culture (non-clonal selection in medium) was isolated and tested for absence of *mafA* and presence of *bla*. Absence of *mafA* and presence of *bla* in NM Δ *mafA* were parameter to select the knock-out clone. Knock-out clone # 3 (bold) was selected for the of hBMECs induction.

SUPPLEMENTARY TABLES

**Supplementary Table S1. Total number of clusters detected and genes mapped**			
Sample		Total number of detected clusters/raw reads	Total number of filtered genes
Neisseria meningitidis	1	12561364	11398
	2	12050257	
	3	11847698	
MafA	1	8953433	11398
	2	10044520	
	3	8016908	

1, 2, 3 present biological replicates

Supplementary Table S2. Primers used in the study

Protein (Gene)	Sequence used to design primers	Primer name	Sequence (5′-3′)	Amplicon length (bp)	Annealing temperature
Primers used to amplify gene coding MafA					
MafA (*mafA*)	AE002098.2*: ntc378274 to ntc377333	MafA F	TATGGATCCGGCAAACGCTTTGCCGTCGAAC	843	60°C
		MafA R	TTTGGTACCGATGACTTCGTTGCCGACATC		
Primers used to confirm insert in transformants					
---	---	UA Insertom F1	CGCATCACCATCACCATCACG	---	60°C
---	---	UA Insertom R1	ACCAAATTGGGACAACACCAGTG		
Primers used for qRT-PCR					
C-X-C motif chemokine ligand 10 (CXCL10)	BC010954.1	CXCL10 F	GCCATTCTGATTTGCTGCCTT	179	55°C
		CXCL10 R	GCAATGATCTCAACACGTGGAC		
Intercellular adhesion molecule 1 (ICAM-1)	BT006854.1	ICAM-1F	CCGGCCAGCTTATACACAAGA	124	60°C
		ICAM-1R	CACATTGGAGTCTGCTGGGAA		
Vascular cell adhesion molecule 1 (VCAM-1)	AK291732.1	VCAM-1F	CCCTGAGCCCTGTGAGTTTT	138	60°C
		VCAM-1R	GGCCACCACTCATCTCGATT		
C-X-C motif chemokine ligand11) (CXCL11)	Y15220.1	CXCL11F	CCCTGGGGTAAAAGCAGTGA	149	60°C
		CXCL11R	GCCTTGCTTGCTTCGATTTG		
Interleukin 6 (IL-6)	M18403.1	IL6F	AAAACAACCTGAACCTTCCA	104	55°C
		IL6R	CTCCAAAAGACCAGTGATGA		
Interleukin 8, C-X-C motif chemokine ligand 8 (IL-8)	BT007067.1	IL8F	CTCCAAACCTTTCCACCCCA	153	55°C
		IL8R	TTCTCCACAACCCTCTGCAC		
TSC22 domain family member 3 (TSC22D3)	AF153603.1	TSC22D3F	GACAACAAGATCGAACAGGCC	107	60°C
		TSC22D3R	TTCTCCACCAGCTCTCGGAT		
TIMP metallopeptidase inhibitor 4 (TIMP4)	AK313018.1	TIMP4F	ACGCCTTTTGACTCTTCCCT	127	60°C
		TIMP4R	AGGGCTCGATGTAGTTGCAC		
Natriuretic peptide receptor 3 (NPR3)	AK300094.1	NPR3F	ACCTGAGTTTGAGAAGTTTTCCATG	122	60°C
		NPR3R	AGACGTAGAGGAGGATGGCA		
Solute carrier family 16 member 12 (SLC16A12)	BC041140.1	SLC16A12F	ATTGTGGCTGGCTGTTTCCT	117	60°C
		SLC16A12R	CCATGCCGTTTGTGCGTAAT		
β–microglobulin (B2M)	XR_002957658.1	B2MF	GCTCGCGCTACTCTCTCTTT	134	55°C

		B2MR	CGGATGGATGAAACCCAGACA		
Primers used to construct a cassette of knock out MafA gene					
Fragment 1	AE002098.2*: ntc376969 to ntc377332	KO MafA-F1 F	TGATTTGGCGGATTTTGATGGG	363	55°C
		KO MafA-F1 R	AGCGGGGTTTGTTTGATGTTG		
Beta-lactamase protein (bla)	pLEXI I ble3**: nt 5543 to nt7493	KO-BLA F	CAACATCAAACAAACCCCGCTATGAGTATTCAACATTTCCGTGTCG	861	55°C
		KO-BLA R	AAGGAGGTTTGTCAGTCTGCGTTACCAATGCTTAATCAGTGAGGC		
Fragment 2	AE002098.2*: ntc378275 to ntc378593	KO MafA-F2 F	CGCAGACTGACAAACCTCCTT	318	55°C
		KO MafA-F2 R	GTCGAAGGGGGCGTGTTCTT		

*shows Genbank accession number followed by nucleotide positions spanning the gene. ** Vector pLEXY I-ble3 (Cat. no. EGE-244; Jena bioscience). Restriction sites are depicted with underlined nucleotides - GGATCC – *BamH*I and GGTACC – *Kpn*I.

SUPPLEMENTARY METHODS

Supplementary method S1

Human brain microvascular endothelial cell (hBMECs) culture. In short, hBMEC/D3 cell line was obtained from Merck/Millipore (Prague, Czech Republic) and cultured in 25-mL cell culture flask coated with collagen type I (Sigma, USA) in EBM-2 medium (Lonza, UK) containing 10% FBS, gentamycin, 1.4 µM hydrocortisone (Sigma), 5 µg/mL ascorbic acid, 10 mM HEPES and 1 ng/mL bFGF (Sigma). Cells were incubated at 37ºC in a humid atmosphere of 5% CO2 until confluence. Cells from the monolayer (6th passage) were used in the experiment.

Supplementary method S2

Selection and culture of clones for recombinant protein production. The gene fragments encoding MafA was amplified by PCR from genomic DNA. Detailed information on primer, amplicon length and restriction enzymes used are presented in **Supplementary Table S2 online**. Amplified fragment was digested with restriction enzymes *BamH*I and *Kpn*I (Thermo Fisher Scientific, Slovakia), as per the manufacturer's instruction. Digested fragments were ligated into pQE-30-mCherry-STOP plasmid (**Supplementary Fig. S1 online**). Please note that in this vector mCherry serves as stuffer sequence, which is cut out during the digestion of vector with restriction enzymes. Ligation mix contained 2µl of ligation buffer, 2µl of PEG8000, 0.5 U of the T4 ligase, amplicon and plasmid (50 ng) in molar ratio of 10:1, and water up to 20 µl. Ligation was performed at 22°C for 1 hr. Ligation mix was purified using NucleoSpin (Macherey-Nagel, Germany) and transformed into *E. coli* M15 strain (Qiagen, Germany). Transformants were selected from LB agar plates (lysogeny broth, Sigma; supplemented with 2% bacteriological agar, 1% glucose, 25 µg/mL kanamycin and 50 µg/mL carbenicillin). Presence of encoding gene in transformants was

confirmed by sequencing (vector specific primers UA Insertom F and R, presented in **supplementary table S2 online**).

A single colony carrying *MafA* gene was cultivated in Terrific broth (TB) (15 g/L tryptone, 30 g/L yeast extract, 12.5 g/L NaCl, 2.5 g/L $MgCl_2$/$MgSO_4$, 100 µL/L metal mix, 7.5 mL/L glycerol) supplemented with 1% glucose, 50 µg/mL carbenicillin and 25 µg/mL kanamycin until OD_{600}= 6. Bacterial cells were pelleted (centrifugation at 6,000 × g for 10 minutes) and resuspended in fresh TB medium without glucose. Protein expression was induced with 1 mM IPTG (Fermentas, Slovakia) at 20 °C for 20 hrs.

Supplementary method S3

Isolation and purification of the MafA. After induction, cells were pelleted (17,880 × g for 10 minutes) and lysed in lysis buffer (0.03 M Na_2HPO_4, 0.5 M NaCl, 0.001% Tween 20, 10% glycerol, 1x protease inhibitor cocktail, Sigma-Aldrich) with four freeze-thaw cycles followed by sonication on ice (2 cycles; 30-s pulses, 100% amplitude). Proteins were purified with nickel affinity chromatography (Ni-NTA agarose beads, ABT agarose Spain) as per manufacturer´s instructions. Briefly, Ni-NTA beads were equilibrated with the buffer (50 mM Na_2HPO_4, 300 mM NaCl, 0.001% Tween 20, pH 8). Beads were then incubated in the lysate for 2 hr at 8 °C with constant rotation (140 rpm). Unbound proteins were washed with washing buffer (50 mM Na_2HPO_4, 300 mM NaCl, 0.001% Tween 20, 20 mM imidazole, pH 8) for 5 times and protein were eluted in elution buffer (50 mM Na_2HPO_4, 300 mM NaCl, 250 mM imidazole, pH 8).

Protein was immediately subjected to gel filtration (Sephadex G25, 30 ml column, *in-house* prepared) on ÄKTApurifier (GE-healthcare, 2 ml/min flow, max 0.45 MPa pressure) to remove imidazole and accomplish the buffer exchange. The buffer used for exchange was 50 mM Na2HPO4 buffer pH 7.0. Protein was immediately processed with cation exchange

chromatography (polishing step) with following conditions on ÄKTApurifier (Ge healthcare): column – Resource S (1ml, GE healthcare), start buffer - 50 mM Na2HPO4 buffer pH 7.0, elution buffer - 50 mM Na_2HPO_4 buffer with 1 M NaCl pH 7.0, flow rate – 1 ml/min, gradient 0% to 100% of elution buffer in 5 CV (column volume). Please note that, scouting was performed to standardize elution conditions described above (scouting of various gradient, salt concentration and CVs). Gradient of 5 CV was kept finally to concentrate the proteins. Chromatograph presenting conditions is in

50 mM Na_2HPO_4 buffer pH 7.0 was added to the eluted protein to adjust the Nacl concentration between 0.1 - 0.2 M and protein was loaded on modified-polylysine resin (high capacity endotoxin removal resin, Thermo-scientific) packed in 1 ml column (*in-house* column packing). Conditions for this chromatography performed on ÄKTApurifier were: both start and elution buffers (isocratic condition) - 25mM sodium phosphate buffer containing 0.1 M NaCl pH 7.0 (prepared in endotoxin free water), flow rate - 0.15 ml/min, pressure limit - 0.25 MPa maximum, sample load - 2 ml. Please note that eluate was collected in biosphere endotoxin free tubes (Sarsted, Germany) and protein was stored immediately in several aliquots until use.

Supplementary method S4

SDS-PAGE and MADLI-TOF

Whole cell lysate of *E.coli*, flow-through from nickel affinity chromatography, MafA eluted from nickel affinity chromatography and MafA eluted after endotoxin removal were separated on SDS-PAGE. Briefly, the protein samples (5 µl each) were mixed with lithium dodecyl sulfate sample buffer (4X LDS sample buffer, Invitrogen, Slovakia) as per manufacturer's instructions and incubated at 72°C for 15 min. Electrophoresis was carried out at 40 mA in 1 x running buffer (20 x NuPAGE MOPS SDS running buffer, Invitrogen) until the dye reached the bottom of the gel

(10% Bis-Tris Polyacrylamid 12 well gel, Invitrogen). Protein were stained with silver staining kit as per manufacturer's instructions (Bio-Rad).

For MALDI-TOF, 0.8 µl of the purified protein (after endotoxin removal) was mixed with 0.8 µl sDHB matrix (Bruker Daltonics, Germany; sDHB dissolved up to saturation in TA50 (50:50 [v/v] acetonitrile : 0.1% TFA in water). 1 µl of the protein-matrix mix was spotted on the ground-steel plate (Bruker Daltonics) and allowed to air dry. Acquisition was performed in flexControl V 3.4 in linear mode with 60 Hz laser intensity (200 shots) on Microflex with reflectron MALDI mass spectrometer (Bruker Daltonics). Mass was analyzed in flexAnalysis V3.4 software of Bruker Daltonics by comparing it with calibrants (protein calibration kit I, Bruker Daltonics).

Supplementary method S5

Construction of the cassette to knock-out the *mafA* gene

Two fragments, 363 bp upstream (fragment 1) and 318 bp downstream (fragment 2) to *mafA* gene, were amplified by PCR from genomic DNA of *N. meningitidis* M1/03. The beta lactamase gene (*bla*) was amplified from the vector pLEXI I ble3 (Jena Bioscience, Germany). Master mixes for amplification each fragment comprised of: 5 µL of High fidelity buffer (Jena Bioscience), 0.5 µL of 12.5 mM dNTP, 0.6 µL of forward primer (25 µM), 0.6 µL of reverse primer (25 µM), 5 µL of DNA template (100-500 ng), 0.5 µL of high fidelity polymerase (1.25 U) and water up to 50 µL. Sequences of the primer are presented in **Supplementary Table S2 online**. Note that, fragment 1, *bla* gene and fragment 2 were fused together with overlap extension PCR (OE-PCR). Primers with overhang were designed to facilitate fusion of 3 fragments to obtain fusion construct ***Fragment 1-bla-Fragment 2*** (details are in **supplementary Fig. S6, panel A)**. For all three fragments cycling conditions were as follows: [95°C for 2 min., 35 cycles of (95°C-2 min., 55°C- 30 sec, 68°C- 1

min.), 68°C- 2 min.]. Amplicons were excised from 0.7% borax agarose gel and purified with NucleoSpin (Macherey-Nagel, Germany).

All three purified fragments were mixed in equimolar concentration (5 nM) in the presence of 2.5 µL of Taq buffer (Jena Bioscience), 0.5 µL of dNTP, 0.2 µL of Taq polymerase (1 U, Jena Bioscience) and water up to 25 µL. Cycling condition for first fusion step was: [95°C-2 min, 11 cycles of (95°C-2 sec, 55°C-30 sec, 72°C-2 min), 72°C-10 min]. PCR product was column purified and subjected for second OE-PCR.

Master mix of second OE-PCR contained: 2.5 µL of amplified product from first OE-PCR, 2.5 µL of Taq buffer, 0.5 µL of dNTP, 0.4 µL of each primer KO-MafA F1 F and KO-MafA F2 R (25 µM), 0.2 µL of Taq polymerase and water up to 25 µL. Cycling conditions were: [95°C-2 min, 31 cycles of (95°C-2 sec, 55°C-30 sec, 72°C-2 min), 72°C-10 min]. Amplicon of the fused fragment was gel purified purified (Macherey-Nagel, Germany) and used for transformation of *Neisseria*.

Please note that, fragment 2-bla-fragment 2 fused cassette is designed to replace mafA gene via homologous crossover.

Supplementary method S6

Transformation of *Neisseria*

N. meningitidis M1/03 was cultured overnight on brain heart infusion agar (BHI agar) supplemented with 10 mM $MgCl_2$. Note that older colonies should not be used for transformation. Three isolated colonies were picked and washed 3 times with 500 µL of 0.3 M sucrose. Finally, bacteria were resuspended in 100 µL of 0.3 M sucrose and transferred into the tube containing 200 ng of dried cassette (produced with OE-PCR). Mixture was incubated for 10 minutes incubation at room temperature and 1 min on ice. Electroporation conditions were: 2 mm gap cuvette, 2.5 kV,

200 Ω and 25 μF in Gene Pulser Xcell (Bio-Rad). Bacteria were resuspended immediately in 1 mL of BHI medium, incubated 6 hours at 37°C in 5% CO_2 atmosphere and then transferred into 5 mL of BHI medium containing carbenicillin (concentration 12.5 μg/mL). After 72 hours 150 μL of culture was plated on BHI agar with carbenicillin (12.5 μg/mL) and incubated at 37°C in 5% CO_2 until single isolated colonies were appeared (48-72 hours).

Four isolated colonies (clones) were picked and each was resuspended in 100 μL of BHI medium. 50 μL from each suspension was inoculated in 5 ml of BHI medium containing carbenicillin. Cultures were grown 24 hours as described above and glycerol stocks were prepared. Remaining 50 μL of the suspension was used to isolate genomic DNA (95°C for 10 min heating). Deletion of *mafA* was confirmed by PCR using *mafA* specific primers as described above. Insertion of *bla* gene was also confirmed by PCR using KO-BLA F and KO-BLA R primers (**Supplementary Table S2 online**).
